# Phylogenetic Tracings of Proteome Size Support the Gradual Accretion of Protein Structural Domains and the Early Origin of Viruses from Primordial Cells

**DOI:** 10.3389/fmicb.2017.01178

**Published:** 2017-06-23

**Authors:** Arshan Nasir, Kyung Mo Kim, Gustavo Caetano-Anollés

**Affiliations:** ^1^Department of Biosciences, COMSATS Institute of Information TechnologyIslamabad, Pakistan; ^2^Evolutionary Bioinformatics Laboratory, Department of Crop Sciences, University of Illinois at Urbana-ChampaignUrbana, IL, United States; ^3^Division of Polar Life Sciences, Korea Polar Research InstituteIncheon, South Korea

**Keywords:** phylogenomics, tree of life, origin of viruses, protein structure, Heaps law, proteome growth

## Abstract

Untangling the origin and evolution of viruses remains a challenging proposition. We recently studied the global distribution of protein domain structures in thousands of completely sequenced viral and cellular proteomes with comparative genomics, phylogenomics, and multidimensional scaling methods. A tree of life describing the evolution of proteomes revealed viruses emerging from the base of the tree as a fourth supergroup of life. A tree of domains indicated an early origin of modern viral lineages from ancient cells that co-existed with the cellular ancestors. However, it was recently argued that the rooting of our trees and the basal placement of viruses was artifactually induced by small genome (proteome) size. Here we show that these claims arise from misunderstanding and misinterpretations of cladistic methodology. Trees are reconstructed unrooted, and thus, their topologies cannot be distorted *a posteriori* by the rooting methodology. Tracing proteome size in trees and multidimensional views of evolutionary relationships as well as tests of leaf stability and exclusion/inclusion of taxa demonstrated that the smallest proteomes were neither attracted toward the root nor caused any topological distortions of the trees. Simulations confirmed that taxa clustering patterns were independent of proteome size and were determined by the presence of known evolutionary relatives in data matrices, highlighting the need for broader taxon sampling in phylogeny reconstruction. Instead, phylogenetic tracings of proteome size revealed a slowdown in innovation of the structural domain vocabulary and four regimes of allometric scaling that reflected a Heaps law. These regimes explained increasing economies of scale in the evolutionary growth and accretion of kernel proteome repertoires of viruses and cellular organisms that resemble growth of human languages with limited vocabulary sizes. Results reconcile dynamic and static views of domain frequency distributions that are consistent with the axiom of spatiotemporal continuity that is tenet of evolutionary thinking.

## Introduction

Untangling the origin and evolution of viruses is one of the most challenging questions in evolutionary biology. Two major competing scenarios have been proposed: (i) viruses are very ancient and evolved (or co-existed) prior to the origin of modern cells, and (ii) viruses evolved recently from genetic material in host cells that “escaped” cellular control and became infectious (reviewed in Claverie, 2006; Forterre, 2006, 2016; Koonin et al., 2006; Bandea, 2009; Holmes, 2011a; Abergel et al., 2015; Nasir et al., 2015). The “virus-early” vs. “virus-late” debate is central to answering some of the toughest questions in biological research such as how and when did life originate on Earth, how to define and treat viruses (are they alive?), did viruses evolve once or multiple times in evolution, and how viruses and cells interact with each other in their bid for survival. Naturally, the topic has remained contentious (Raoult and Forterre, 2008; Claverie and Ogata, 2009; Koonin et al., 2009; Moreira and Lopez-Garcia, 2009; Claverie and Abergel, 2013, 2016).

The deep evolutionary exploration of viral origins however is often impossible with traditional phylogenetic and sequence-recognition methods (e.g., BLAST) due to the relatively higher mutation rates of viral genes that can lead to mutational saturation of genomic sequences (Krupovic and Bamford, 2011; Abrescia et al., 2012). This is well known among structural biologists who have shown that viral lineages infecting distantly related hosts sometimes exhibit strong morphological and three-dimensional (3D) similarities in capsid and coat protein structural components of virions, even in the presence of negligible sequence similarities (Benson et al., 2004; Abrescia et al., 2012). We therefore embarked on a large-scale data-driven study of the origins and evolution of viruses (Nasir and Caetano-Anollés, 2015) taking full advantage of the conservation of protein structure over long evolutionary timespans (Chothia and Lesk, 1986; Illergård et al., 2009; Caetano-Anollés and Nasir, 2012; Lundin et al., 2012). We studied the evolution of protein fold superfamilies (FSFs), as defined by the Structural Classification of Proteins (SCOP) database, which include protein domains harboring common structural cores and biochemical functions indicative of a common origin (Andreeva et al., 2008; Fox et al., 2014). FSF domains are not subject to the effects of non-orthologous replacement and lineage sorting by sequence polymorphisms (Philippe and Laurent, 1998; Kim and Caetano-Anollés, 2012). In addition, only a small proportion of FSF domains (i.e., between 0.4 and 4% in Gough, 2005) might have experienced convergent evolution including horizontal gene transfer (HGT). FSF domains are thus evolutionarily highly conserved and represent reliable markers to explore deep evolutionary relationships (Nasir et al., 2012a).

Our large-scale analysis utilized a combination of comparative genomics, phylogenomics, and multidimensional scaling methods to study the evolution of a *total* of 1,995 FSF domain structures in ~11 million proteins from 5,080 proteomes sampled from 1,420 cellular organisms and 3,460 viruses from the seven known viral replicon types (Nasir and Caetano-Anollés, 2015). The most parsimonious interpretation of our data strongly supported the virus-early scenario of viral evolution, indicating that viral lineages originated multiple times in evolution (i.e., in a polyphyletic manner) from ancient cells (either by primordial reduction or escape; Forterre and Krupovic, 2012; Nasir et al., 2012b; Nasir and Caetano-Anollés, 2015) that predated and/or co-existed with the early ancestors of superkingdoms Archaea (A), Bacteria (B), and Eukarya (E). However, the study disfavored the possibility of viral origins prior to the “first cell” (i.e., the virus-first scenario, Koonin et al., 2006) because viruses by definition must reproduce in an intracellular environment and because the early co-existence of viral and cellular ancestors was supported by several lines of evidence, including:

- A cohort of 442 *universal* (i.e., ABEV) FSFs out of *total* 1,995 (22%) that was enriched in ancient proteins associated with cell membranes and appeared first as a group in a timeline of FSFs derived from a phylogenomic tree of domains (ToD). The ABEV domains suggested an early cell-like existence in the history of modern viruses.
- A core of 68 FSFs common to viruses infecting Archaea (i.e., archaeoviruses), Bacteria (bacterioviruses), and Eukarya (eukaryoviruses) (hereafter the V_*abe*_ group, Table S1) indicating that these viral lineages existed prior to the diversification of cellular life.
- The abundance of virus-specific proteins lacking any homologs in cellular proteomes (>75% putative viral ORFans) that endowed unique identity to the viral supergroup (V).
- The reconstruction of phylogenomic trees (and networks) that placed viruses at the base of a rooted tree of life (ToL).
- An evolutionary principal coordinate (evoPCO) analysis projecting a “four-domain” view of cellular and viral proteomes rooted in evolutionary and geological time (Nasir and Caetano-Anollés, 2015).

We also ruled out the virus-late scenario because it implies little or no genetic overlap among archaeoviruses, bacterioviruses, and eukaryoviruses, an assumption shown to be false by structural studies (Bamford, 2003; Benson et al., 2004; Abrescia et al., 2012) and the existence of the V_*abe*_ group of FSF domains (Table S1; Nasir and Caetano-Anollés, 2015).

Recently, Harish et al. (2016) criticized our phylogenomic methods and the virus-early scenario claiming that the basal position of viruses in our ToLs was due to a so-called “small genome attraction” (SGA) artifact attracting viruses (and other organisms) encoding small-sized proteomes toward the base of the rooted ToLs. Two of the authors are proponents of an origin of life in Eukarya and previously reconstructed a very complex most recent universal common ancestor of life encoding ~75% of the total protein folds known today (Harish et al., 2013). Their proposal, which goes counter to modern evolutionary thinking, relies on an evolutionary model that penalizes protein domain gains three times over losses (3:1), violates the “triangle inequality” property of phylogenetic distances needed for valid phylogenetic optimization, and produces an “upside down” phylogeny that attracts organisms with large genomes such as plants and animals to the base of their ToL (see Kim et al., 2014 for a discussion of these shortcomings). Here, we objectively address the criticism of a proteome size-induced basal placement of viral and prokaryotic proteomes. We show that Harish et al. (2016) confused key concepts of our phylogenomic methodology (summarized in Table 1), including our rooting methodology and character polarization scheme, the meaning of “genome size,” and downplayed “rules of thumb” for taxa selection in genome content and composition-based phylogenies. Importantly, they missed the crucial fact that our phylogenomic trees are reconstructed unrooted, and thus, their topologies cannot be distorted *a posteriori* by the rooting methodology, as claimed by Harish et al. (2016). Here we make explicit that the basal placement of viral and prokaryotic proteomes in our trees represents the *modus operandi* of long-term evolutionary processes of gene gains and losses that result in the gradual accretion of structural domains and the collective growth of proteomes over evolutionary time. While both gains and losses frequently participate in proteome evolution, their systematic phylogenetic tracing on a ToL indicated that gains significantly outnumbered losses (80,904 gains vs. 47,848 losses in Nasir et al., 2014b), especially in prokaryotic proteomes. Because there are several ways to gain proteins (e.g., HGT, *de novo* gene creation, and neo/sub-functionalization following gene duplication) relative to losing them (e.g., gene loss as a one-time irreversible event), numerically gains override losses resulting in gradual accretion of domains and proteome growth (Nasir et al., 2014b). This complex interplay extends to the viral supergroup and results in universal scaling patterns, which are discovered by phylogenomic reconstructions but cannot be predicted by the effects of ill-defined proxies of “genome size.”

**Table 1:** Fact-checking the narrative of Harish et al. (2016).

**Fiction (Harish et al., 2016)**	**Fact**
“*A re-examination* of Nasir and Caetano-Anollés' *phylogenomic approach suggests that small genomes systematically distort their phylogenetic reconstructions*.”	In their re-examination, Harish et al. (2016) reconstructed trees (their Figures 1, 2) without paying attention to the rooting, character polarization, and taxa sampling details of our phylogenomic methodology. To exacerbate, they added extreme examples of cellular endosymbionts that complicate the definition of valid taxa in phylogenetic reconstructions.
We “*use a hypothetical (ancestor) pseudo-outgroup,” “a hypothetical ancestor,”* or “*…an artificial ‘all-zero’ taxon*…*an ‘all-absent’ hypothetical ancestor”* to root the ToL, or “*an ancestor that is assumed to be an empty set of protein domains”* as outgroup to “*create specific phylogenetic artifacts*.”	Outgroups indicate sister taxa external to the ingroup (the taxon set being studied), which are defined *a priori* as being of more ancestral nature. Unless taxa describe either resurrected or *in vitro* evolved molecules or microbes in long-term evolution experiments (e.g., artificial phylogenies, Hillis et al., 1992), outgroups are never ancestors. They are typically extant taxa, which are *a priori* assumed to form one of two separate convex groups together with the ingroup. No outgroup taxon (presumably extant, hypothetical or artificial) was ever used or defined in our study or used as an ancestor (Nasir and Caetano-Anollés, 2015). Furthermore, we do not combine outgroups and ancestors, an approach known to be invalid (Bryant, 1997).
“*Including the hypothetical ancestor during tree estimation amounts to a priori character polarization*.”	We polarize character transformations *a posteriori*, empirically and most parsimoniously, and complying with Weston's generality criterion (Weston, 1988, 1994).
“*Unrooted trees describe relatedness of taxa based on graded compositional similarities of characters*.”	The search of tree space using maximum parsimony as an optimality criterion is defined by homology relationships manifesting in tree branches not graded compositional similarities.
“*Accordingly, we can expect the ‘all-zero’ ancestor to cluster among genomes (proteomes) in which the smallest number of superfamilies is present. The latter are the proteomes described by the largest number of “0s” in the data matrix.”*	During phylogenetic searches, we first optimize character change in unrooted trees using the Wagner algorithm (Farris, 1970). The topology of rooted trees cannot be predicted from patterns in character state vectors of ingroup or outgroup taxa and thus cannot be affected by genome size.
“*Including viruses in the analyses draws the root toward the smaller viral proteomes.”*	A simple node distance (*nd*) vs. genome size plot dispels their putative SGA artifact for viruses (Figure 4). Contrary to their claim, including viruses decreases overall tree instability (Figure 8, Table 2).
“*Half of the sampled proteomes were analyzed (Figures 1, 2) for computational simplicity.”*	They included only 16 eukaryal (not 17 as they claim), 17 archaeal, 17 bacterial, and 5-9 viral proteomes, which only represent ~16% of our taxa and likely missed representation of key phyla/groups in their trees (Nasir and Caetano-Anollés, 2015). Trees are not comparable.
“*The exclusion of highly reduced ‘parasitic’ proteomes appears to be inconsistent with the inclusion of viruses.”*	Our exclusion and inclusion of taxa followed clear rationale. Exclusion of organisms engaged in obligate cellular endosymbiosis ensured integrity of definition of taxa. Inclusion of representatives of all viral groups portrayed the entire viral supergroup, which is unified by its parasitic lifestyle.
“*Small proteome size is not an irreconcilable feature of genome-tree reconstructions.”*	The article referred by the authors (Harish et al., 2013) has resulted in the reconstruction of a very complex most recent common ancestor of cells encoding almost 75% of existing protein folds. Two of the authors are proponents of an origin of Eukarya (and highly complex organisms) at the base of the ToL, which goes against modern evolutionary thinking. Their phylogenomic method uses polarized characters with arbitrary transformation costs, which violate the “triangle inequality” of phylogenetic distances and are engineered to attract large genomes to the base of their trees. Their use of unrealistic evolutionary assumptions does have irreconcilable consequences for the correct reconstruction of trees (Kim et al., 2014).
“*49 of 68 core-SFs are unique to dsDNA viruses and 32 of these are found in Mimivirus genes. The latter are known to be acquired by cell-to-virus HGT, either from the host amoeba or from bacteria that parasitize the host amoeba.”*	All 49 core-FSFs (i.e., V_*abe*_ FSFs common to archaeoviruses, bacterioviruses, and eukaryoviruses) are found in mimiviruses (Table S1). The majority of core-FSFs are indeed commonly detected in dsDNA viruses as hitherto no RNA viruses are known to infect Archaea and are rare in Bacteria (Nasir et al., 2014a; Koonin et al., 2015). They further stated that core-FSFs were acquired by viruses from their cellular hosts, specifically belonging to Acanthamoeba. However, core-FSFs are by definition not restricted to dsDNA viruses of Eukarya but are widespread among archaeoviruses and bacterioviruses. The argument about possible horizontal acquisition of core FSFs from amoeba or bacterial hosts is highly speculative and goes against recent bioinformatics explorations revealing an abundance of virus-specific genes lacking cellular homologs (Daubin et al., 2003; Cortez et al., 2009). Furthermore, the authors do not provide any evidence to support their statements. Core-FSFs do not cross the superkingdom barrier to infect eukaryotic hosts (e.g., a total of 10,427 instances of core-FSFs were detected in bacterioviruses compared to 5,823 in eukaryoviruses, Table S1). Virus transfers between superkingdoms have never been observed either in nature or the laboratory (Forterre, 2016).
“*Likewise, their supporting data and analyses seem to be biased by limited sampling and highly skewed superfamily distributions. Indeed, the data presented here undermine the inferred relative antiquity of viruses in the ToL.”*	To compare, our genomic dataset included 5,080 proteomes of 3,460 viruses and 1,620 cells in comparison to their inclusion of only 9 viruses and 51 cells (their Figures 1, 2). Clearly, Harish et al. (2016) performed limited sampling and explored highly skewed FSF distributions.
“*The instability of rooting with an all-zero ancestor becomes clear when the smallest proteome in a given taxon sampling varies in the rooting experiments.”*	Harish et al. (2016) misunderstood the rooting methodology, confused stability of rooting with leaf stability, and did not report tree metrics of any kind to test the validity of their trees. They wrongly labeled one of the two most basal bacteria (taxid: 262724) an as archaeon (their Figure 1B). They selected taxa with larger genomes than those we sampled (their Figure 2D). Thus, genome size cannot be the culprit of the alleged tree distortions since our trees harbor smaller genomes and are stable. Instead and unsurprisingly, their choice of adding rogue taxa destabilized their phylogenies.

*A somehow similar table can be found in a eLetter exchange (Nasir and Caetano-Anollés, 2015)*.

## Results and discussion

### A brief overview of structural phylogenomics methodology

There are several pre-processing steps involved in the reconstruction of rooted phylogenies to ensure maximum protection from biological and technical artifacts. First, *taxa* are sampled broadly while ensuring participation from each major group of organisms (and viruses) since increased taxon sampling is known to decrease phylogenetic error (Heath et al., 2008). Taxa are distinguished by the “profile” distribution of molecular characters, which in this case represent abundance (i.e., *reuse*) of FSF domains in sampled taxa. Data matrices are then processed to remove group-specific FSFs (e.g., the large number of eukaryote-specific immunoglobulin FSFs lacking counterparts in prokaryotic and viral proteomes) and FSFs with zero abundance. These filtering steps reduce the data matrix to comprise only of *universal* (i.e., ABEV) FSFs to increase resolution in the deep branches of the ToL. Data matrices are then transformed and normalized to an alpha-numeric scale indicating 24 (or 32 or 64) possible character states (e.g., 0–9 and A–N) representing FSF abundances in sampled taxa. These matrices are imported into the PAUP^*^ software for phylogeny reconstruction (Swofford, 2002). During searches of tree space and *prior to rooting*, we optimize character changes in *unrooted* trees allowing for both increases and decreases in FSF abundance (e.g., see gains vs. loss tracings in Nasir et al., 2014b). The resulting most parsimonious unrooted trees that are retained are then rooted using the Lundberg approach (Lundberg, 1972; i.e., *a posteriori*), which still preserves the optimized topology. Thus, *tree topology is established prior to rooting and theoretically cannot be distorted by genome size* (see empirical data discussed below), which is a property of taxa (i.e., proteomes) and not individual characters (i.e., FSFs) changing on trees. In other words, our tree building methodology precludes the systematic SGA artifacts proposed by Harish et al. (2016) because decreasing proteome size decreases the number of contributed phylogenetic characters, not how character states change during phylogenetic reconstruction.

### Rooting trees of life (ToLs): outgroup vs. generality criterion

**Figure 1:**
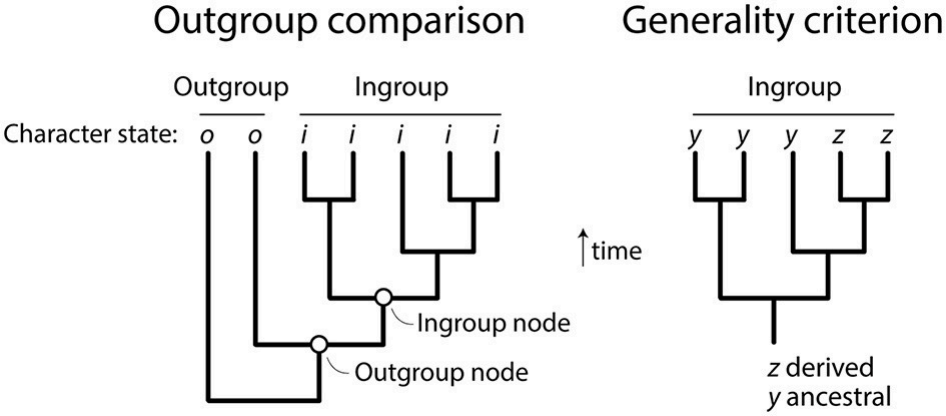
Comparing the indirect outgroup comparison method of rooting trees and the direct generality criterion. Rooting involves orienting an unrooted tree and pulling down a branch that will hold the ancestor of all taxa examined. In outgroup comparison, sister (outgroup) taxa external to the study group (ingroup taxa) are identified *a priori* of being of ancestral origin and the branch that is closest to the ingroup pulled down. This creates a new outgroup node for rooting the phylogeny. The outgroup node adds a character state vector that includes character state *o*, which is diagnostic of the outgroup and is assumed to be ancestral and absent in the ingroup. Once the outgroup is made ancestral, the tree is rooted and character state *i* is shared and derived, making it a synapomorphy. In Weston's generality criterion (Weston, 1988, 1994), the character state distributions in the phylogeny are used to polarize character transformations. Character state *z* is less distributed than *y* within the ingroup (it is present only in a minority subset of taxa) and is considered shared and derived. The figure was modified from Bryant (2001).

Contrary to the claims of Harish et al. (2016), our rooting approach does not involve any outgroup taxon presumably extant, hypothetical, artificial, or treated as an ancestor (see Table 1). Therefore, the indirectly rooted ToLs they build using their “*hypothetical ‘all-zero’ ancestor”* do not mimic or undermine our methods (Figures 1, 2 in Harish et al., 2016). Their tree searches were also conducted differently and with the undesirable property of being dependent on the location of the root. In contrast, we minimize Farris' *f*-values, a measure of the goodness-of-fit of the matrix of path length distances to the matrix of original distances, which describes total pairwise homoplasy and is independent on the location of the root (Farris, 1972).

To clarify, the rooting method we applied is grounded in early and well-established cladistic formalizations (Farris, 1970; Lundberg, 1972) and is *direct* because it polarizes character transformations with information solely present in ingroup taxa, distinguishing ancestral from derived character states (Figure 1). Character polarization is only applied empirically and *a posteriori* to root the trees: *(a)* considering character spread in nested branches while accounting unproblematically for homoplasy, *(b)* searching for the most parsimonious solutions out of the two possible polarization schemes of the ordered characters while treating homologies as taxic hypotheses, and *(c)* allowing both gradual and punctuated build-up of evolutionary emergence of protein structures, including gain and loss, that complies with the principle of spatiotemporal continuity, Leibniz's *lex continui* (Leibniz, 1687). Trees are rooted using Weston's generality criterion (Weston, 1988, 1994), which states that as long as ancestral characters are preponderantly retained in descendants, ancestral character states will always be more generic than their derivatives given their nested hierarchical distribution in rooted phylogenies (Figure 1). Biologically, protein domain structures spread in evolution when genes duplicate and diversify, genomes rearrange, and genetic information is exchanged. This is a process of accumulation and retention of iterative homologies, such as serial homologs in morphology and paralogous genes in genomes (Weston, 1994), which is global, universal and largely unaffected by proteome size. This same process is widely used to generate rooted phylogenies from paralogous gene sequences. The Lundberg method (Lundberg, 1972), which does not attach outgroup taxa to the ingroup as Harish et al. (2016) claim, simply enables rooting by the generality criterion (Bryant, 1997).

Weston's rule was repeatedly validated by inverse polarization (Felsenstein, 1983) of our ordered (Wagner) characters, which always produced suboptimal trees (e.g., Figures 3, 4 in Kim et al., 2014). In contrast, Harish et al. (2016) did not take into account that rooting is not a neutral procedure. While the length of the most parsimonious trees is unaffected by the position of the root, making *a priori* polarization unnecessary (Farris, 1970), rooting impacts the homology statements of the undirected networks (Lundberg, 1972). “*The length of a tree is unaffected by the position of the root but is certainly not unaffected by the inclusion of a root”* (Brower and de Pinna, 2012). Importantly, Harish et al. (2016) did not report tree metrics, making their tree reconstructions open to speculative interpretations. Wheeler (2012) made it clear: “*For trees to participate in hypothesis testing, we must be able to evaluate them and determine their relative quality. In order to do this, we require a comparable index of merit.”* Generally this comes in the form of a cost or some other objective function based on data and tree. “*Without such a cost, trees are mere pictures—‘tree-shaped-objects’ of no use to science”* (Wheeler, 2012).

### Limitations of taxon sampling and use of Ill-defined genome size proxies

**Figure 2:**
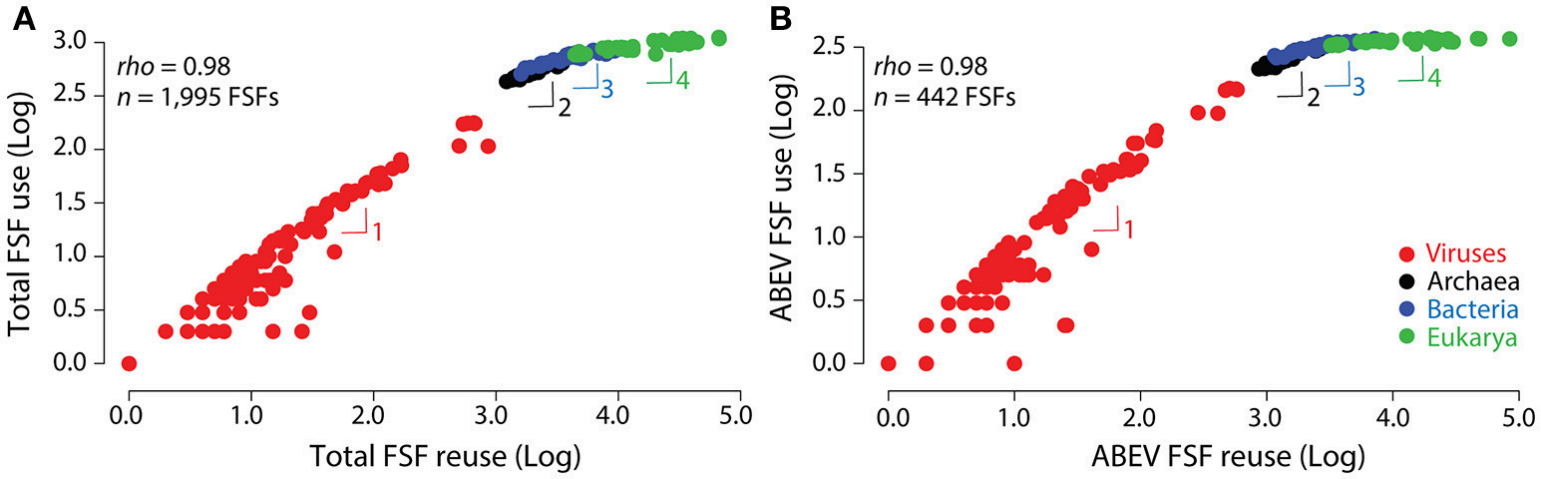
FSF *use* (occurrence) and *reuse* (abundance) are strongly correlated. Scatter log-log plots reveal a strong correlation between FSF *use* and FSF *reuse* for *total* **(A)** and *universal* ABEV FSF **(B)** sets for 368-taxon trees (Nasir and Caetano-Anollés, 2015). Viruses (266), Archaea (34), Bacteria (34), and Eukarya (34) are colored red, black, blue, and green, respectively. Each of these supergroups has its own power law regime that complies with a four-regime Heaps law of vocabulary growth. Individual regimes are indicated with numbers and follow *V* ~ *N*^β^ relationships, with *V* representing FSF vocabulary size (*use*) and *N* representing FSF database size (*reuse*) in proteomes. Their fits to linear regression models using ordinary least squares and the estimation of the Heaps exponent β are described in Figure S2.

Harish et al. (2016) claimed that “genome size” defined by the total number of distinct FSFs encoded by each genome (i.e., FSF occurrence that we here term FSF *use*) was the determinant of taxa positions in their rooted 60-taxon ToLs (representing subsets of our 368-taxon trees in Nasir and Caetano-Anollés, 2015). They argued that organisms encoding small-sized genomes clustered together leading to topological distortions and caused mixing of taxa from different superkingdoms. It is important to first note differences between the two experimental designs before we address the existence of the alleged SGA artifact:

- *Taxon sampling:* Our 368-taxon ToL described evolutionary relationships of an equal number of Archaea, Bacteria, and Eukarya (34 each) and at least 5 viruses from each known viral family/order (a total of 266 viruses belonging to 87 ICTV families) (Nasir and Caetano-Anollés, 2015). These trees included each major phyla/group in the same proportion that was present in the original 5,080-dataset comprising 1,620 cellular organisms and 3,460 viruses. In comparison, Harish et al. (2016) extracted 17 species each from Archaea, Bacteria, and Eukarya, and only 9 viruses from our data matrix to produce 60-taxon trees without explaining any taxon selection rationale. Absence of close-relatives in trees could lead to unrealistic and arbitrary groupings and topological distortions that increase phylogenetic error (Heath et al., 2008), as observed in the 60-taxon trees of Harish et al. (2016) but not in our 368-taxon trees (**Figure 7** in Nasir and Caetano-Anollés, 2015) or even in Harish et al. trees (Figure S3 in Harish et al., 2016) when they restored the full taxon cellular set.
- *Genome size definition:* Genome size cannot be defined by FSF *use* when exploring a putative SGA artifact because our phylogenomic data matrices build evolutionary trees from FSF *reuse* (i.e., abundance or redundant count of FSFs in taxa). In other words, a single FSF could be present multiple times in the same genome owing to well-known evolutionary processes such as gene duplication, amplification and HGT (Nasir et al., 2014b), their multiplicity contributing to overall genome size. Moreover, organisms that are related by a relatively recent common ancestor will likely have similar FSF abundance profiles compared to organisms separated by large evolutionary distances (emphasizing the need for broader and inclusive taxon sampling). In addition, gene loss and reductive evolution, which can occur both in free-living and parasitic/obligate parasitic organisms (and viruses) (Dufresne et al., 2005; McCutcheon and von Dohlen, 2011), can decrease FSF *use*. The interplay between FSF *use* (the domain vocabulary) and FSF *reuse* (the proteomic use of the domain vocabulary) of *total* (i.e., the entire repertoire) or *universal* (i.e., ABEV) FSFs contributes meaningful information to our data matrices (Figure 2) and *neither of the two alone can define genome size for predicting taxa placement in trees*. Thus, Harish et al. (2016) definition of genome size is ill defined.
- *Universal characters:* Only *universal* ABEV FSFs were kept in the phylogenomic data matrix for tree reconstruction purposes (Nasir and Caetano-Anollés, 2015). Although *use* and *reuse* of *total* and *universal* FSFs are positively and strongly correlated, indicating a link between protein fold innovation and abundance (Figure 2), there are interesting and significant differences. For example, *Emiliania huxleyi* encodes a total of 963 FSFs, out of which 378 (39%) are ABEV (Table S2). This organism has the highest number of distinct *universal* FSFs among all sampled eukaryotes, even greater than *Mus musculus* (370 FSFs) and *Homo sapiens* (369). However, in terms of *total* FSFs, *E. huxleyi* encodes the 11th “largest” proteome in eukaryotes harboring 963 FSFs (Table S2). Similarly, the bacterium *Sorangium cellulosum* encodes 371 distinct *universal* FSFs, exceeding the ABEV *use* of all eukaryotic proteomes except *E. huxleyi* (Table S2). Because it is the *universal* set, and specifically FSF *reuse*, that is included in the phylogenomic data matrix, defining organism genome size by *total* FSF *use* (or even ABEV *use*; Harish et al., 2016) would be incorrect. Furthermore, we observed lack of correlation between ABEV FSF *use* and genome size for cellular organisms (Figure S1), which indicates that using total FSF *use* as extrapolation of our *universal* FSF set is a misleading proxy for genome size.

### No “small genome attraction” (SGA) artifact

**Figure 3:**
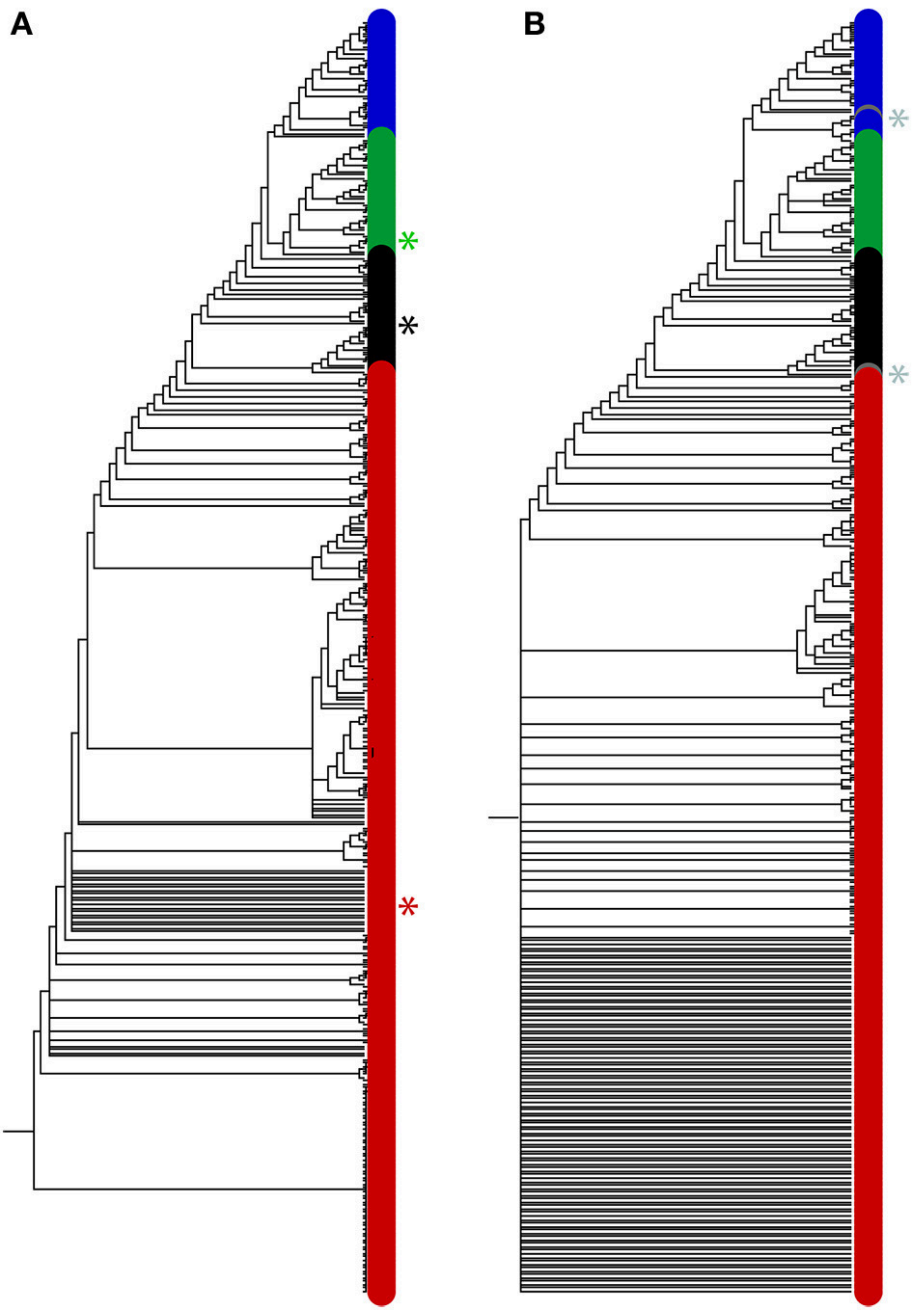
Trees of proteomes are robust and insensitive to the effects of genome size but sensitive to holobiont relationships defining taxa. **(A)** The single most parsimonious tree (taxa = 368; characters = 442; length = 45,935, retention index = 0.83, *g*_1_ = −0.31) describing the evolution of 102 cellular organisms (34 each from Archaea, Bacteria, and Eukarya) and 266 viruses (sampled at least 5 viruses from each family/order) (Nasir and Caetano-Anollés, 2015). The smallest proteomes for cells (*I. hospitalis* and *A. gossypii*; black and green asterisks) and viruses (bat cycloviruses; red asterisk) are indicated. The names of taxa are not shown because they would not be visible. Instead, the positions of terminals were colored according to supergroup, green (Eukarya), blue (Bacteria), black (Archaea) and red (viruses). **(B)** A strict consensus of two most parsimonious trees (length = 46,781, retention index = 0.83, *g*_1_ = −19.81 and −19.82) built using phylogenomic data from the 368 proteomes of panel **(A)** plus the proteomes from the two extremely reduced *R. prowazekii* and *N. equitans* (gray circles and asterisks). While no major topological distortions are observed, the consensus tree losses resolution at its base.

Our 368-taxon ToL (Figure 3A) dissected organisms and viruses into four supergroups (see also Figure 7 in Nasir and Caetano-Anollés, 2015). Importantly, there was no mixing of taxa from different supergroups in the ToL despite of considerable overlap in FSF *use* and *reuse*, especially among cellular organisms (Figure 2, examples above). The ToL revealed that taxa recognized their true evolutionary relatives thanks to the complex interplay between FSF *use* and *reuse*, which acts as composite variable (e.g., an archaeon encoding 100 FSFs will still be distinguished from a bacterium encoding 100 FSFs as the two organisms will likely have different FSF *reuse* and will also differ in the composition of the 100-FSF set). Labeling the phylogenetic positions of the “smallest” proteomes in our trees (defined by ABEV FSF *use* and *reuse*) confirmed that the smallest genomes were not attracted toward the root. For example, among the 102-cellular taxa used in our ToL (Figure 3A), the euryarchaeote *Ignicoccus hospitalis* was the smallest proteome either by *universal* FSF *use* (*n* = 213 ABEV FSFs) or *reuse* (868). The archaeon however did not appear at the root of the cellular subtree but appeared at a rather well derived position within the archaeal subtree (Figure 3A, see the black asterisk). Even the smallest virus in our dataset (the 1.7 kb bat cyclovirus encoding a single FSF and harboring a ssDNA genome) did not appear with basal RNA viruses but clustered with its closest evolutionary relative, the Dragonfly cyclovirus at the more derived positions (Figure 3A, red asterisk). Similarly, *Ashbya gossypii* was the smallest eukaryotic proteome (*use* = 326 FSFs, *reuse* = 3,217 FSFs) but was not the most basal eukaryote within the eukaryal subtree (the most basal was *Cyanidioschyzon merolae, use* = 331, *reuse* = 3,507), although it appeared in basal positions (Figure 3A, green asterisk). In turn, the bacterial proteome with lowest FSF *use* (*Lactobacillus delbrueckii*, 261 FSFs) was not the smallest with FSF *reuse* (*Aquifex aeolicus*, 1,155 FSFs).

Importantly, topological distortions do not appear in our ToLs (Figure 3) despite FSF *use-reuse* value overlaps (Figure 2) negating the existence of proteome-size dependent taxa clustering. This is showcased by the observation that the addition of the extremely reduced proteomes of *Rickettsia prowazeki* (Bacteria, *use* = 201, *reuse* = 626) and *Nanoarchaeum equitans* (Archaea, *use* = 131, *reuse* = 345) that caused topological distortions and mixing of archaeal and bacteria taxa in the 60-taxon trees of Harish et al. (2016) had no such effects on either the crown of 368-taxon trees (Figure 3B) or even when Harish et al. (2016) restored the full taxon set of 102 cellular organisms (Figure S3 in Harish et al., 2016). We emphasize that Harish et al. (2016) did not increase sampling of viral taxa from 9 to 266. It is interesting to note that *N. equitans* encodes a proteome even smaller than some “giant” viruses such as *Acanthamoeba polyphaga mimivirus* (*use* = 149, *reuse* = 508) and *Megavirus chilensis* (146, 581) but does not cause any distortions by mixing with viral taxa. The exercise therefore confirms that the smallest proteomes do not “fight” for the basal positions in trees. Instead, they recognize their true evolutionary relatives during exhaustive tree optimization of information in ABEV FSF *use* and *reuse* values that are encoded in the evolutionary data matrix. The addition of *R*. *prowazekii* and *N. equitans* however reduced support of phylogenetic relationships at the base of our ToLs (Figure 3B), an expected outcome when adding “rogue” taxa known to assume varying positions in sets of optimal trees (Thorley and Wilkinson, 1999). In brief, the ToLs strongly negate arbitrary groupings of taxa based on genome size.

**Figure 4:**
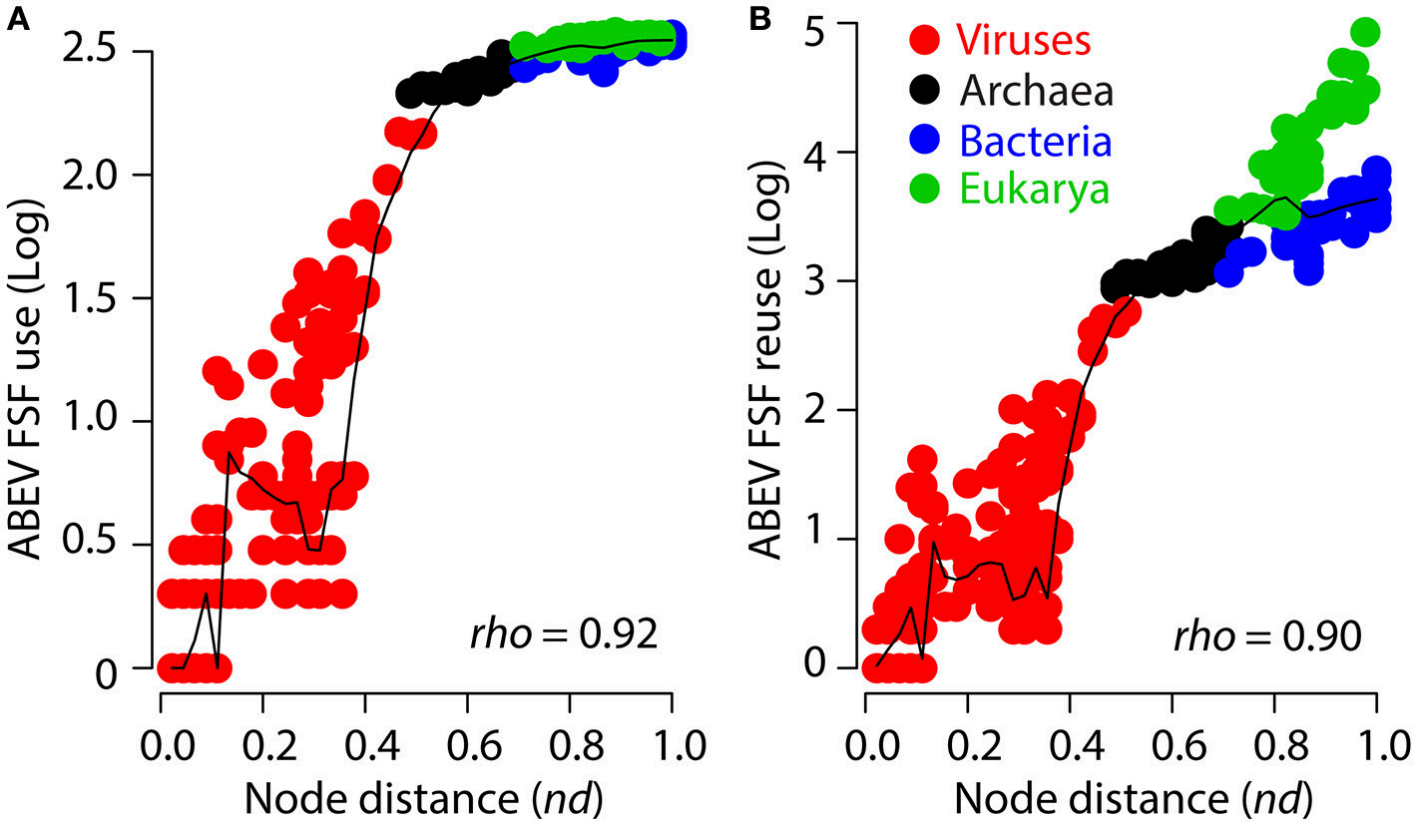
Scatter plots describe the relationship between ABEV FSF *use* **(A)** and *reuse* **(B)** and node distance (*nd*) for the 368-taxon ToL (Nasir and Caetano-Anollés, 2015). Data points for different supergroups are colored green (Eukarya), blue (Bacteria), black (Archaea) and red (viruses). The black line describes the nature of the relationship, as determined by the Locally Weighted Regression Scatter Plot Smoothing (LOWESS) method, which obtains a smoothed curve by fitting successive regression functions (*q* = 0.1, *i* = 100). The plot reveals high scatter, especially toward smaller *nd* values and clustering of bacterial and eukaryal taxa in the same *nd* range despite harboring big differences in FSF *use* and *reuse*.

To empirically demonstrate the absence of a systemic SGA artifact, we plotted the “node distance” (*nd*) from the root to each terminal node (i.e., taxa) of the ToL—on a scale from 0 (most basal) to 1 (most recent)—against ABEV FSF *use* and *reuse* of supergroup taxa (Figure 4). The *nd* variable describes on a relative scale how evolutionarily derived is each taxon in the tree. The plots revealed substantial scatter, especially in viruses, and genome-size independent clustering of cellular proteomes indicating an absence of systemic SGA (Figure 4). For example, despite comparable FSF *use-reuse* between archaeal and bacterial proteomes, bacterial proteomes occupied a similar *nd* range with eukaryotic proteomes albeit harboring big differences between their *use-reuse* values (see also different slopes between Bacteria and Eukarya in Figure 2). However, a generic tendency of increase in proteome growth (mediated by both gains and losses of FSF domains throughout the evolutionary timeline, Nasir et al., 2014b) is obvious but reflects the strong link between protein fold innovation and abundance (i.e., FSF *use-reuse*) that exists for both viral and cellular proteomes and is discovered by our reconstructions. For example, many bacterial proteomes overlap archaeal proteomes in FSF *use* and *reuse*, and so do many bacterial and eukaryal proteomes (Figure 4). However, their placement in the trees is at well-derived positions and comparable to eukaryotic taxa rather than archaeal taxa with their lower *nd* values.

**Figure 5:**
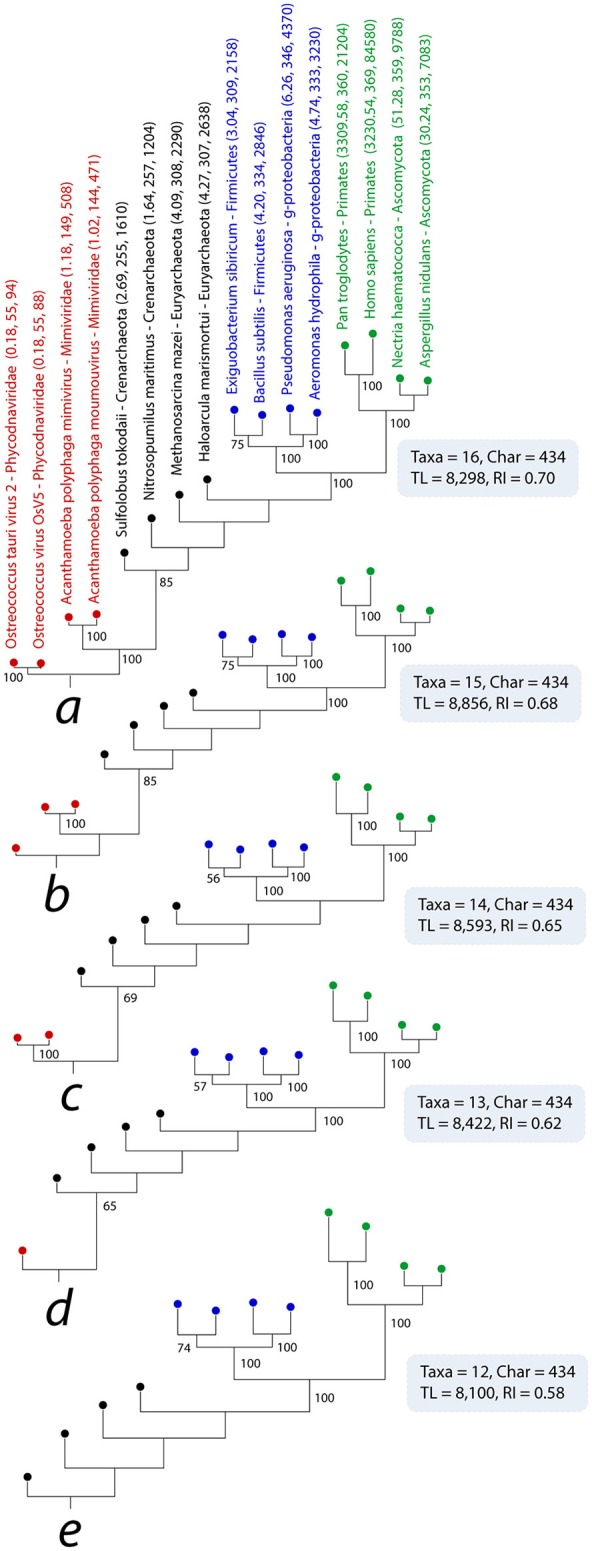
Testing the SGA artifact with the Siddal and Whiting (1999) approach. A single most parsimonious phylogenomic tree (*a*) describes the evolutionary relationships between four proteomes sampled each from viruses, Archaea, Bacteria, and Eukarya. Taxa are colored as previously described. Numbers on branches indicated BS support values (%). Single most parsimonious trees *b* through *e* were recovered after successive elimination of the smallest viral proteomes. TL, tree length; RI, retention index.

Next, we performed a simple test for the existence of the alleged SGA that was inspired by the Siddal and Whiting test of the long branch attraction (LBA) artifact (Siddal and Whiting, 1999). The test evaluates clades influenced by putative LBA by removing (for example) one of the two long branched taxa from the phylogenetic tree. Under LBA, such removals are expected to change the topology of the tree, as the branch attracted to the putative long branch is now free to occupy its correct phylogenetic position (reviewed in Bergsten, 2005). To extrapolate this logic, if a small-sized genome attracts another small-sized genome, then removal of the offending genome will restore the attracted genome to its accurate (different) phylogenetic position on the tree. To test, we selected 2 primates and 2 ascomycetes from Eukarya, 2 Crenarchaeota and 2 Euryarchaeota from Archaea, 2 Gamma-proteobacteria and 2 Firmicutes from Bacteria, and 2 mimiviridae and 2 phycodnaviridae from viruses (the 4444 dataset). We intentionally kept organisms and viruses of known taxonomies in the data matrix to observe any topological distortions influenced by taxa removal during tree reconstructions. Taxa were labeled both by *use* and *reuse* of ABEV FSFs (Figure 5). In the first reconstruction, we recovered the four-supergroup ToL without any topological mixing (Figure 5, tree *a*). Remarkably, FSF *use* and *reuse* of *Exiguobacterium sibiricum* (Firmicute) were either comparable or significantly lower to the *use* and *reuse* of the two euryarchaeotes included in the tree (309 and 2,158 vs. 307 and 2,638 and 308 and 2,290), respectively. Still, *E. sibiricum* clustered with its Firmicute relative, *Bacilus subtilis*, with good bootstrap (BS) support (72%). Nevertheless, applying the Siddal and Whiting test, we next removed the smallest viral proteomes sequentially, *Ostreococcus tauri virus 2, Ostreococcus tauri virus OsV5, Acanthamoeba polyphaga moumovirus*, and *Acanthamoeba polyphaga mimivirus* (Figure 5, trees *b* through *e*). None of the exclusions changed either the clustering patterns or tree topology indicating that the alleged SGA did not exist and that clustering of viral and prokaryotic proteomes toward the root of the ToL resulted from character change (FSF abundance) optimization in trees, not from properties of the ill-defined genome size.

### Taxon definitions and leaf stabilities prompt exclusion of cellular endosymbionts and inclusion of viruses in ToLs

Our practice of excluding cellular endosymbionts was interpreted as avoidance of genome size attraction artifacts (Harish et al., 2016), when in reality our intention was to exclude organisms with ill-defined hologenomes of holobiont collectives (the host and its associated organismal communities), which are known to complicate definitions of taxa (Zilber-Rosenberg and Rosenberg, 2008; Keeling, 2011). No such exclusion was extended to the viral supergroup since one hallmark of viruses is harboring a life cycle with strict dependence on a cellular host (see below). We previously confirmed that cellular endosymbionts and obligate parasites harbor an FSF domain repertoire that is distinct from the other members of their respective superkingdoms (Nasir et al., 2011). Cellular organisms committed to obligate parasitism show an increase in informational domains that is sometimes offset by loss of metabolic domains. This unique signature is conserved among nearly all known endosymbionts (Nasir et al., 2011) and distinguishes these organisms from other members of their respective superkingdom. The existence of two unique signature FSF repertoires in cellular organisms (i.e., of free-living organisms and endosymbionts) creates conflict when the two lifestyles are considered together in genome-composition phylogenies. It leads to distortions when endosymbionts from different superkingdoms cluster together irrespective of their taxonomic affiliation). In turn, there are no “free-living” viruses and this conflict does not exist in the virosphere.

**Figure 6:**
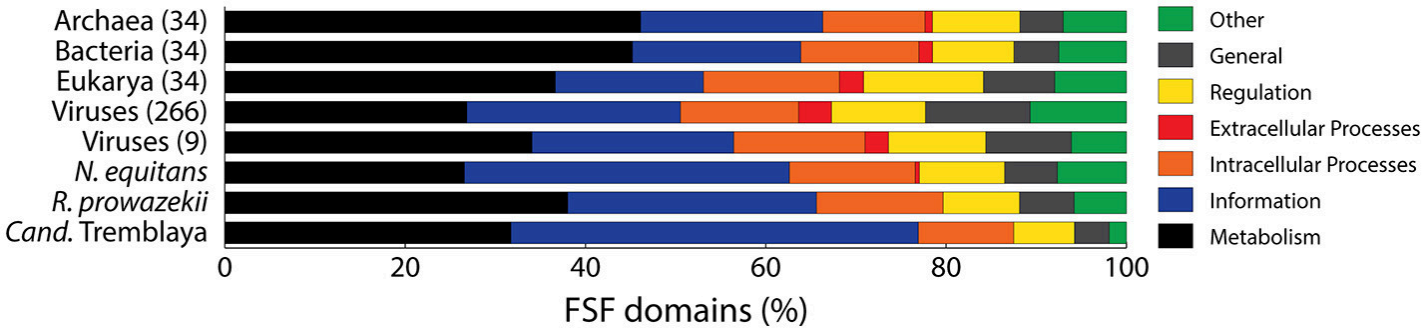
Cellular endosymbionts differ from free-living organisms and viruses in their FSF composition profiles. Annotation of FSF domains into one of the seven major functional categories (*Metabolism, Information, Intracellular Processes, Extracellular Processes, Regulation, General*, and *Other*) for archaeal, bacterial, eukaryal, and viral proteomes sampled in our study (Nasir and Caetano-Anollés, 2015) and for nine viral and three extremely reduced cellular proteomes included by Harish et al. (2016) in their reconstructions *Cand*. Nausia deltocephalinicola was not part of our reconstructions (encodes only 55 *universal* FSFs). Obligate endosymbionts or parasites often increase the repertoire of informational FSF domains, as showcased by *Cand*. Tremblaya included by Harish et al. (2016), and for 311 other known obligate and facultative parasitic organisms in (Figure 3 in Nasir et al., 2011). Functional scheme as defined by Christine Vogel in SUPERFAMILY database (http://supfam.org/SUPERFAMILY/function.html). Category *Other* includes proteins with either unknown or viral functions. *General* includes proteins involved in binding to small molecules, ligands, and lipids, and structural proteins. Numbers in parenthesis indicate total number of proteomes included in the FSF profile representation.

Viruses are also different from cellular endosymbionts in their FSF composition profile (Figure 6) and hence do not cause any distortions to the cellular subtrees (Figure 3). Harish et al. (2016) disregarded the rationale and added questionable taxa to their data matrices. These taxa were likely “cherry-picked” from extreme proteomic outliers and sometimes even outside our initial sampling (e.g., *Cand*. Nausia deltocephalinicola). For example, *Cand*. Tremblaya princeps included in their trees (Figure 2 in Harish et al., 2016) is part of a three-pronged endosymbiotic organismal system (McCutcheon and von Dohlen, 2011). Its genome encodes only 55 *universal* FSFs. It is not considered an independent organism since it depends on its host (*Planococcus citri*) and its endosymbiont (*Cand*. Moranella endobia) to synthesize essential metabolites (López-Madrigal et al., 2011). Similarly, *Cand*. N. deltocephalinicola is an obligate endosymbiont of leafhoppers, which harbors the smallest known bacterial genome (Bennett and Moran, 2013) and encodes only 53 *universal* FSFs. These extreme proteomic outliers do not bias tree reconstructions because of their genome size nor induce “*grossly erroneous rootings,”* as suggested by Harish et al. (2016). Instead, their hologenomes arise from relatively modern genomic exchanges and recruitments likely resulting from complex trade-off relationships that complicate the dissection of their evolutionary origin and their definition as single valid taxon in the phylogenetic data matrices. Phylogenetically, they represent problematic taxa that should be excluded from analysis pending further understanding of their genetic makeup. The intentional inclusion of problematic taxa is expected to generate biased reconstructions (e.g., see Wilkinson et al., 2000 for a dinosaur phylogeny example and the detection of problematic taxa with double decay analysis).

**Figure 7:**
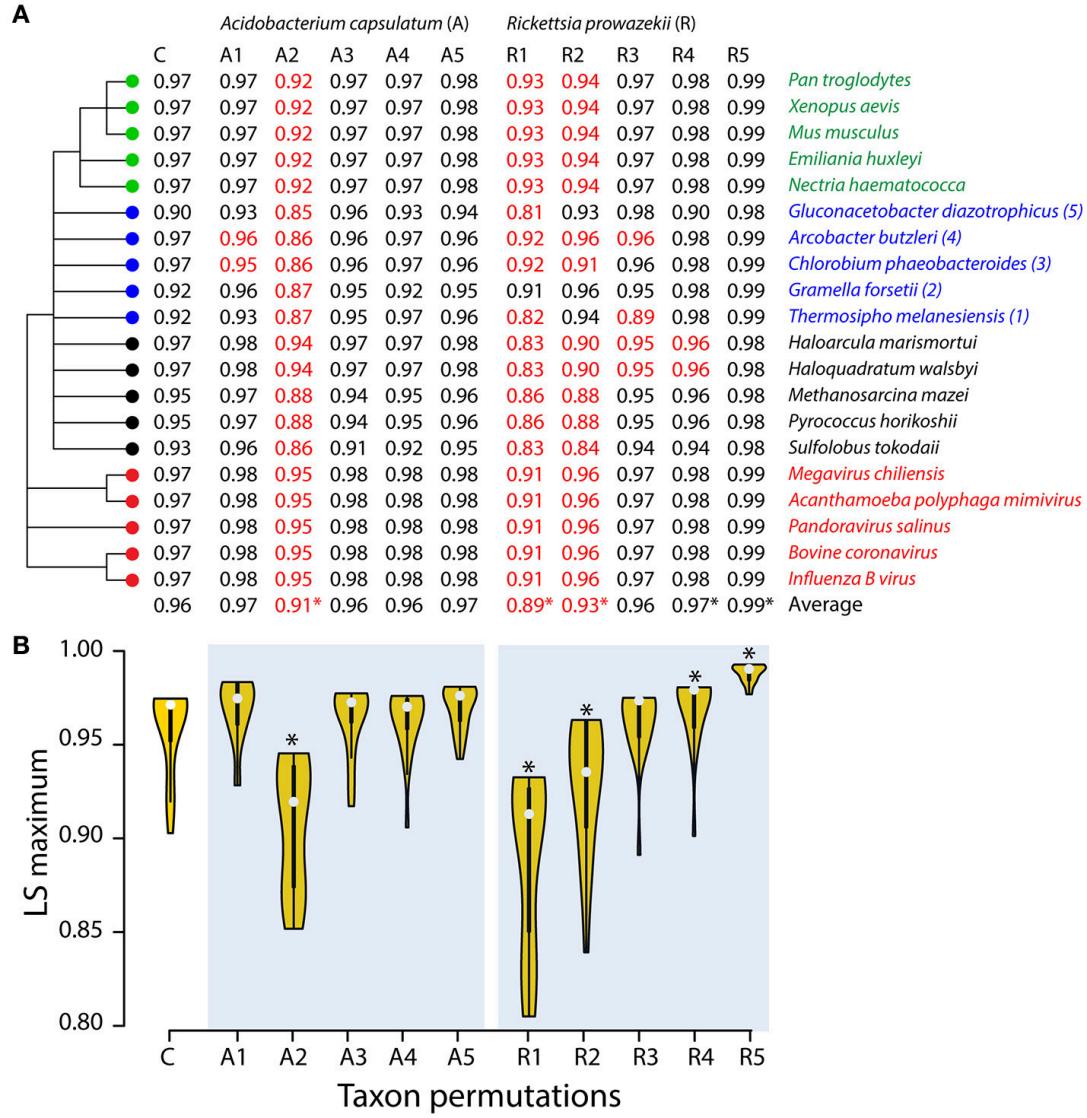
Obligate parasitic taxa destabilize leaves of trees. **(A)** Leaf stabilities (LS maximum) were calculated with RadCon (Thorley and Page, 2000) from 2,000 unrooted BS trees. LS values are ordered in the table **(A)** according to the most informative strict reduced consensus (SRC) tree (33.54 bits) out of a set of 5 SRC trees, which matches the strict component consensus (consensus efficiency = 0.555) derived from the unrooted trees. **(B)** LS values are visualized as violin plots. Violin plot is a combination of the box plot (the black rectangle with white circle representing group median) and density plot on each side (yellow) reflecting data distribution. The spread of LS values was calculated for the control set (C) and all possible permutations of free-living *Acidobacterium capsulatum* (A1–A5) and the obligate endoparasite *R. prowazekii* (R1–R5) with individual taxa of the corresponding bacterial superkingdoms (identified with numbers following taxon labels). The density trace is plotted symmetrically around the boxplots. White circles are group medians. Asterisks are distributions significantly different from control C (Wilcoxon rank sum test, two-tailed, *P* < 0.01).

In the absence of tree statistics, it is impossible to evaluate the effect of progressive inclusion of extremely-reduced obligate parasitic taxa on the reconstructions of Harish et al. (2016). We therefore performed a series of tests to determine if “rogue” taxon addition affected the support of unrooted phylogenies (Figure 7, Table S3). In unrooted trees, the smallest phylogenetic statement is the relationship of a quartet of leaves. When examining BS-resampled phylogenies, the frequency of alternative resolved quartets provides measures of support for the position of each leaf and the accuracy of the tree (Thorley and Wilkinson, 1999). These BS-based leaf stability (LS) indices describe phylogenetic instabilities that often result from either insufficient samplings or conflicting data. Since the genomic census is exhaustive, the culprit of LS varying scores can be character incongruence imposed by problems in the definition of taxa and characters. An unstable leaf can lower the LS scores of the other leaves and affect the overall LS of the taxon set by either occurring in unstable quartets (direct effects) or by lowering the stability of quartets in which it does not occur (indirect effects) when there is character conflict. Figure 7A shows a 20-taxon strict consensus tree with equal representation of supergroup taxa from 2,000 BS replicates used as a control (C). BS replicates were also generated for all 5 possible permutations of the free-living *Acidobacterium capsulatum* control and the obligate endoparasite *R. prowazekii* with the taxon set of the corresponding bacterial supergroup. These replicates were used to evaluate LS measures (Figure 7B, Table S3). Remarkably, LS indices from *R. prowazekii* permutations were significantly more variable and globally lower than those of *A. capsulatum*, explaining the reduced support of phylogenetic relationships we observed at the base of our ToL when the obligate parasites were added (Figure 3B). Similar results were obtained when alternative tree statistics such as LS difference and LS entropy were compared (Table S3) indicating the potentially “rogue” *R. prowazekii* taxon could be excluded from tree reconstructions for better and reliable recovery of evolutionary relationships. Explicitly Agree (EA) similarity, the proportion of quartets including the leaf that are resolved and of the same type in the trees, describe the similarity of the position of leaves (Estabrook, 1992). EA values increase with the putatively rogue *R. prowazekii* taxon (Table S3). Thus, their addition decreases leaf stability while at the same time resulting in similar leaf positions. Finally, the RogueNaRok algorithm (Aberer et al., 2013) also indicated that the *R. prowazekii* taxon was rogue and was a candidate for pruning.

**Figure 8:**
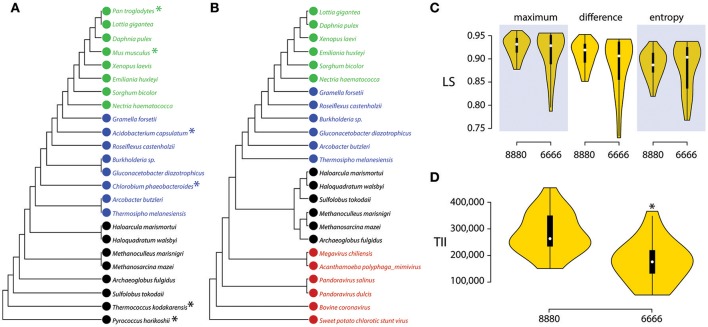
Viruses stabilize leaves of trees. **(A)** A single most parsimonious phylogenomic tree (length = 13,004, retention index = 0.61) reconstructed from the genomic abundance census of 442 *universal* FSFs (432 parsimony informative characters) in 24 proteomes selected equally from Archaea (black), Bacteria (blue), and Eukarya (green) (the 8880 dataset). The most stable taxa in each superkingdoms, as indicated by TII values (Table 2), are labeled with an asterisk. **(B)** A single most parsimonious phylogenomic tree (length = 12,033, retention index = 0.70) reconstructed from the genomic abundance census of 442 *universal* FSFs (428 parsimony informative characters) in 24 proteomes selected equally from viruses (red), Archaea (black), Bacteria (blue), and Eukarya (green) after replacing the most stable cellular taxa in **(A)** with viruses (the 6666 dataset). **(C)** A comparison of various LS statistics between the 8880 and 6666 BS tree datasets, as displayed by violin plots. None of the comparisons were statistically significant (Wilcoxon rank sum test, two-tailed). **(D)** Comparison of TII distribution for the 8880 dataset against the 6666 dataset, as displayed by violin plots. Inclusion of viral taxa significantly reduces overall tree instability. Asterisk indicates significant mean difference (Wilcoxon rank sum test, two-tailed, *P* < 0.01).

Given that the persistence of viruses as a supergroup depends on viral interactions with cellular hosts, considerations of lifestyle and taxon definition alone cannot be used to exclude viruses in phylogenomic reconstructions. Cellular dependency is a necessary condition for the propagation of all viruses (with no exceptions), which generally occurs through lysis, exocytosis and transport (Nasir et al., 2017). Viruses can also engage in host-specific dependency and dormancy interactions via symbiosis and latency (e.g., polydnaviruses and wasps behaving as holobionts; Federici and Bigot, 2003). However, cellular dependencies could result in viruses acting as rogue taxa in phylogenetic reconstructions. We therefore tested the impact of including viruses on the stability of ToL topologies. Figure 8 shows that the reconstruction of 24-taxon unrooted BS trees with 8 taxa each for Archaea, Bacteria and Eukarya, but no viruses (the dataset 8880, Figure 8A) had LS indices that were not significantly different (LS_maximum_, *P* = 0.98 LS_difference_, *P* = 0.61; LS_entropy_, *P* = 0.60) from those where the most “stable” cellular organisms were replaced by 6 viral taxa to produce a balanced 4-supergroup BS set (dataset 6666, Figure 8B). Thus, LS distributions show that viruses and cellular organisms are equally stable in ToLs (Figure 8C). To further inspect the two BS tree sets, we measured taxon instability indices (TII), which compute the variation of pair-wise patristic distances between taxon pairs across all trees (Maddison and Maddison, 2001). TII also evaluates leaf stabilities and the impact of rogue taxa (Aberer et al., 2013). Figure 8D shows that the 8880 unrooted BS trees gain a 37% significant decrease (*P* < 0.01) in taxonomic instability by replacements with the balanced 6666 BS set (Table 2). In addition, none of the viruses that were added were considered rogue taxa and candidates for pruning by the RogueNaRok algorithm (Aberer et al., 2013). Therefore, and contrary to the claims of Harish et al. (2016), phylogenetic stability provides one more reason to include viruses in ToLs.

**Table 2:** Inclusion of viral taxa decreases tree instability.

**8880**		**6666**		**Decrease (%)**
**Taxon**	**TII**	**Taxon**	**TII**	
***Acidobacterium capsulatum***	339984.56	***Acanthamoeba polyphaga mimivirus***	176059.76	–
*Archaeoglobus fulgidus*	275171.68	*Archaeoglobus fulgidus*	276206.40	−0.004
*Arcobacter butzleri*	389177.39	*Arcobacter butzleri*	260465.25	33.07
*Burkholderia* sp.	384956.57	*Burkholderia* sp.	202131.54	47.49
***Chlorobium phaeobacteroides***	348612.99	***Bovine coronavirus***	139006.79	–
*Daphnia pulex*	296748.86	*Daphnia pulex*	51657.62	82.59
*Emiliania huxleyi*	252024.48	*Emiliania huxleyi*	86941.27	65.50
*Gluconacetobacter diazotrophicus*	351756.39	*Gluconacetobacter diazotrophicus*	208054.12	40.85
*Gramella forsetii*	367648.99	*Gramella forsetii*	172381.65	53.11
*Haloarcula marismortui*	245672.98	*Haloarcula marismortui*	227995.40	7.20
*Haloquadratum walsbyi*	244554.08	*Haloquadratum walsbyi*	227292.68	7.06
*Lottia gigantea*	244638.31	*Lottia gigantea*	51716.23	78.86
*Methanoculleus marisnigri*	216223.26	*Methanoculleus marisnigri*	193300.26	10.60
*Methanosarcina mazei*	218019.48	*Methanosarcina mazei*	194245.12	10.90
***Mus musculus***	221980.99	***Megavirus chilensis***	176092.12	–
*Nectria haematococca*	278079.67	*Nectria haematococca*	115236.83	58.56
***Pan troglodytes***	239186.33	***Pandoravirus dulcis***	145974.04	–
***Pyrococcus horikoshii***	151131.73	***Pandoravirus salinus***	143402.03	–
*Roseiflexus castenholzii*	454093.65	*Roseiflexus castenholzii*	216056.64	52.42
*Sorghum bicolor*	271355.69	*Sorghum bicolor*	102020.45	62.40
*Sulfolobus tokodaii*	208389.88	*Sulfolobus tokodaii*	365974.42	−75.62
***Thermococcus kodakarensis***	151131.73	***Sweet potato chlorotic stunt virus***	139006.79	–
*Thermosipho melanesiensis*	350181.51	*Thermosipho melanesiensis*	308662.81	11.86
*Xenopus laevis*	254966.77	*Xenopus laevis*	63294.76	75.18

*Comparison of TII values for the “8880” BS tree dataset with taxa comprising 8 proteomes each from Archaea, Bacteria, and Eukarya against the “6666” that includes 6 proteomes each from Archaea, Bacteria, Eukarya, and viruses. For the construction of the 6666 dataset, two most stable taxa from each of Archaea, Bacteria, and Eukarya were replaced with viral proteomes (highlighted in bold) used by Harish et al. (2016) in their trees. The last column indicates percentage decrease when comparing TII for the 8880 against the 6666 dataset and is only meaningful for unchanged taxa in both experiments*.

### Multidimensional scaling challenges the SGA artifact but supports the gradual evolutionary accretion of structural domains in proteomes

**Figure 9:**
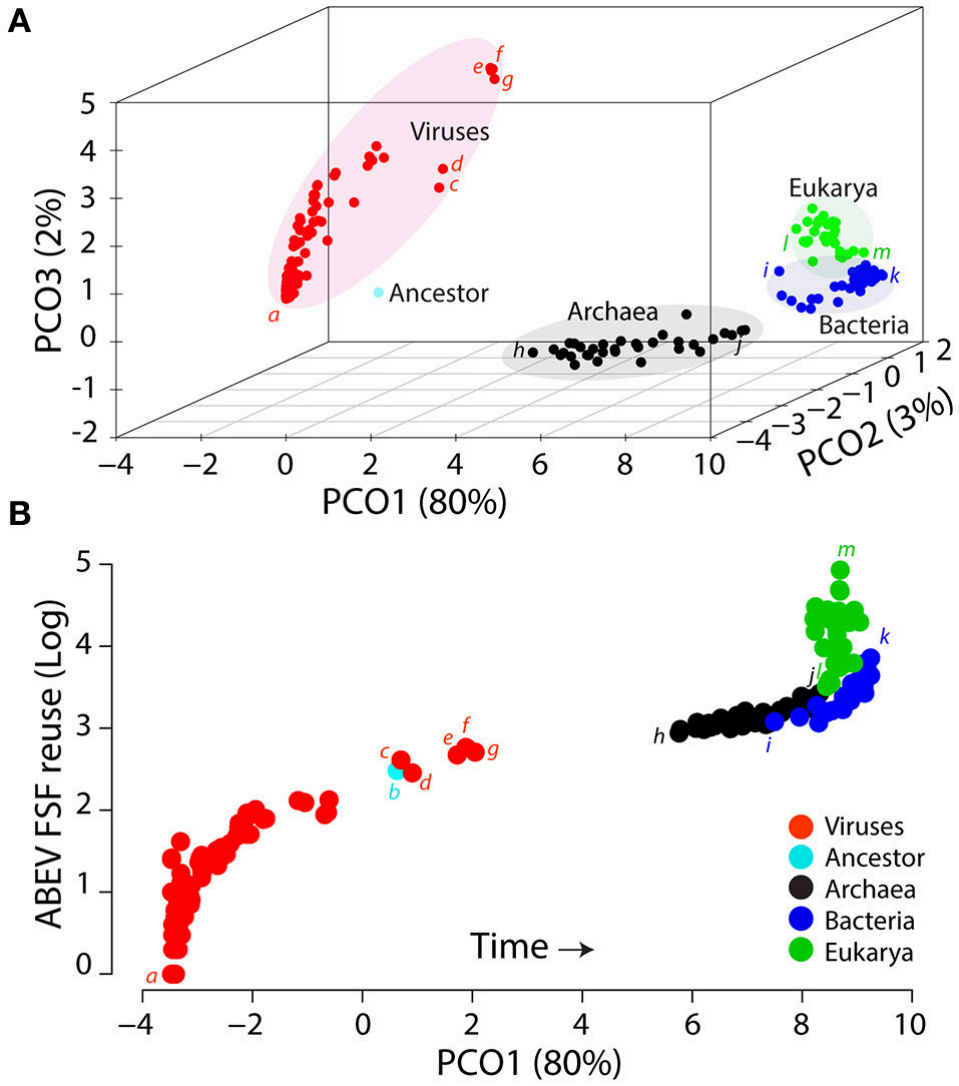
The space of ages of FSF structural domains reveals supergroups as distinct clouds and global evolutionary tendencies of growth in proteomes. **(A)** An evolutionary principal coordinate (evoPCO) analysis plot portrays in its first three axes (85% variability explained) the evolutionary distances between cellular and viral proteomes [taxa = 368, characters = 442 *universal* FSFs, character states = occurrence ^*^ (1−*nd*)]. **(B)** The most important evoPCO component plotted against *universal* ABEV FSF *reuse* in logarithm scale. The reconstructed proteome of the last common ancestor of modern cells was added as reference to infer the direction of evolutionary change (Kim and Caetano-Anollés, 2011). *a*, Lassa virus; *b*, Ancestor; c, *Pandoravisus salinus*; *d, Pandoravirus dulcis*; *e, Acanthamoeba polyphaga mimivirus*; *f*, *Megavirus chilensis*; *g, Megavirus iba*; *h, Ignicoccus hospitalis*; *i, Haloarcula marismortui*; *j, Lactobacillus delbrueckii*; *k, Sorangium cellulosum*; *l, Ashbya gossypii*; *m, Emiliana huxleyi*.

In addition to comparative genomics and phylogenomics data matrices, the virus-early evolutionary scenario was also supported by a 3D evolutionary projection of viral and cellular proteomes treated as biological systems (Figure 8 in Nasir and Caetano-Anollés, 2015). The overall age of each system is determined by the ages of its individual component parts (FSFs, in this case) derived from a ToD describing the evolution of FSFs, which was previously linked to the geological record through a molecular clock of protein folds (Wang et al., 2011). The evoPCO analysis combines the power of cladistics and phenetics and produces a multidimensional view of evolutionary relationships among molecular systems such as proteomes. There are two main advantages of evoPCO: (i) there is no genome-size related variable in the data matrix as FSF abundances are replaced by their relative ages (i.e., evolutionary origin of the FSFs as inferred from *nd* or timelines calibrated in billions of years), and (ii) the method ensures that the fundamental assumption of character independence in phylogenetic tree reconstruction remains intact (Huelsenbeck and Nielsen, 1999; Nasir and Caetano-Anollés, 2015). Figure 9A shows an evoPCO analysis plot explaining in its first three major axes 85% variability in evolutionary distances between 368 cellular and viral proteomes. The plot revealed four distinct temporal clouds of proteomes for viruses, Archaea, Bacteria and Eukarya (Figure 9) and considerable scatter, with patterns resembling those of the *nd* plots previously described (Figure 4). For example, the “*Megavirales*” group (*nd* = 0.44–0.51) with the largest viral proteomes was clearly dissected from the main viral cloud. Its terminal placement suggests the late appearance of “giant viruses” (La Scola et al., 2003; Philippe et al., 2013; Legendre et al., 2014, 2015) in evolution. The placement of supergroup clouds in the evoPCO plot relative to the proteome of the last universal common ancestor of cells reconstructed from Kim and Caetano-Anollés (2011) provided time directionality in the plot, which supported the early rise of viruses, followed by Archaea and a group of Bacteria and Eukarya, in that order. This matches evolutionary patterns of the rooted ToL (Figure 3) and indicates that the topology of the ToL is not due to an artifact induced by genome size because the evoPCO plot relies exclusively on the individual ages of the universal ABEV FSFs of proteomes. These ages cannot be distorted by an SGA artifact since they are derived from a ToD, a phylogenomic tree that describes the evolution of individual structural domains.

To confirm, we studied global patterns of accretion of structural domains by tracing proteome size in the evoPCO analysis plot for each major axis. Figure 9B shows the most important evoPCO component (responsible for 80% of variation) plotted against ABEV FSF *reuse*. We found a gradual increase of the genome size proxy as one travels in time through each axis of the temporal clouds. This confirms that the global tendencies of genome growth we have observed arise from the evolutionary accretion of novel structural domains in the protein world. This is in line with the prevalence of domain gains over domain losses derived from character state reconstructions along the branches of ToLs that describe proteome evolution (Nasir et al., 2014b).

### The heaps law of language and the evolutionary growth of proteome size

While linguistic metaphors have dominated molecular biology since the discovery of DNA, there are striking similarities in the complexity of natural human languages and those of protein and nucleic acid macromolecules (Searls, 2002). This has prompted the use of linguistic theory to explain the modular makeup of proteins (Gimona, 2006). For example, the combination of structural domains in multi-domain proteins resembles the combination of atomic linguistic units (morphemes) that form higher-level units such as words or phrases (lexemes). Remarkably, protein structure complies with a number of language laws, most prominently the Zipf law, the statistical paradigm of linguistics (Zipf, 1949). The Zipf law is a power law that links the rank of a word with its frequency. This link can be presented as a probability density distribution *P*(*k*) ~ *k*^−γ^, where *P*(*k*) is the probability that a word be present *j* times in a text and γ is an exponent that approximates 2. The Zipf law explains patterns of occurrence of Pfam domains in proteins that match words in Shakespeare's *Romeo and Juliet* (Searls, 2002). The law is a special case of the scale-free distribution that it explains, which pervades the rich-get-richer behavior of connections in many biological networks, including those describing metabolism and protein-protein interactions (Barabási, 2009). The Zipf law is followed by structural domains at fold and FSF levels (Qian et al., 2001; Caetano-Anolles and Caetano-Anollés, 2003), with γ decay values of ~2 for Bacteria and Archaea and ~1.4 for Eukarya (Caetano-Anolles and Caetano-Anollés, 2003) matching values for the English and Chinese languages, respectively (Li et al., 2016). Domain structure is also subject to functional type laws that link two kinds of variables. The combination of domains in mutidomain proteins follows the Menzerath-Altmann (MA) law of language distilled by the motto: “*the greater the whole, the smaller its constituents”* (Shahzad et al., 2015). The law governs the size of domains in proteins and expresses a diminishing return tendency associated with trade-offs between economy of matter-energy and information in domain makeup. Both, the Zipf and MA laws describe “principles of least effort” that lessen costs of communication or information in any system.

We now show that the FSF *use* and *reuse* plots of Figure 2 comply with another important law that links language properties to time, with time expressed as accumulating innovation, the Heaps law. This law describes how vocabulary sizes (*V*) are concave increasing power laws of text database sizes *N*, with *V* ~ *N*^β^, where β represents the Heaps exponent (Heaps, 1978). The signature of the law is sublinear growth (β < 1), which is typical of “economies of scale” showing increasingly marginal returns for new vocabulary innovations. Note that the Heaps law can be interpreted in the context of a Zipf distribution when β = 1/γ, that this relationship has been empirically confirmed under asymptotic conditions, that vocabulary and database size are proportional to time, and that constituents of vocabularies can be constant over centuries if they represent “kernel” words that appear with high frequency (Petersen et al., 2012; Gerlach and Altmann, 2013). These properties have interesting implications for proteome growth. For example, the study of deviations in tail distributions linked to the Heap law regression can estimate if a pan-genome representing a gene or domain core shared between a group of organisms will continue to expand when more genomes are explored, defining “open” or “closed” pangenomic repertoires (Tettelin et al., 2005; Koehorst et al., 2016). When these tail distribution deviations were offset in a study of a large body of English text, Ferrer i Cancho and Solé (2001) discovered that the probability density function showed two scaling regimes. The steepest regime followed a Zipf law characterizing a “kernel” lexicon of frequently used words. The other regime characterized an “unlimited” lexicon of growing words of less frequent use. The two-regime Zipf distribution translates into a two-regime Heaps law with β exponents close to 1 for the kernel and 0.4–0.7 for the unlimited lexicon of a number of Indo-European languages, with exponent variation reflecting differences in language organization. These regimes showcase a decreasing marginal need for new words and a slowdown (cooling) of linguistic evolution (Petersen et al., 2012). Recent studies of languages with limited dictionary sizes such as Chinese, Japanese, and Korean (Petersen et al., 2012; Lü et al., 2013) have shown multi-regime Heaps laws. A recent study shows Chinese text follows a 3-regime Heaps law with β scaling exponents of 1, 0.7, and 0.3 for increasing text lengths, which is explained by a stochastic feedback model of vocabulary growth driven by two probabilities, one for the reuse of frequently used words and the other for the rise of word novelties (Li et al., 2016).

Remarkably, the FSF *use* and *reuse* log-log plots of Figure 2 show not two but four distinct power law patterns suggestive of four regimes of slowdown of vocabulary growth, each corresponding to the proteomes of viruses, Archaea, Bacteria and Eukarya, in that order (fittings in log-log plots are shown in Figure S2). Table 3 describes how the vocabulary of *total* and ABEV FSF domains scales with corresponding proteomic datasets with decreasing β, ranging from exponents of ~1 for viruses to approximating 0 for Eukarya (Figure 4). Thus, viral proteomes use a very ancient kernel-like vocabulary with β exponents of 0.81 approaching unity but not far from the second regime of languages with limited vocabularies (β = 0.7–0.77, Petersen et al., 2012; Lü et al., 2013). This ancestral kernel is then expanded successively by growing vocabularies with slowdowns in the proteomes of Archaea and Bacteria and to an extreme in the proteomes of Eukarya, as these gradually appeared in evolution. The values of β for the proteomes of Archaea (β = 0.36–0.40) are not far away from those of English text corpora (β = 0.4–0.7), such as the Gutenberg Project e-book collection (β = 0.45, Tria et al., 2014). The values of β for the proteomes of Bacteria (β = 0.19–0.26) match those of the third regime of Chinese language (Petersen et al., 2012; Li et al., 2016).

**Table 3:** Scaling exponents summarizing the Heaps law for the four distinct regimes that correspond to viruses and the cellular superkingdoms (see also Figure S2).

**FSF set**	**Regime**	**β**	**R^2^**	***F***	***P*-value**
ABEV	1-Viruses	0.81	0.94	4,243	2.2E-16
	2-Archaea	0.36	0.83	160	5.5E-14
	3- Bacteria	0.19	0.89	259	2.2E-16
	4-Eukarya	0.03	0.49	32	2.8E-6
Total	1-Viruses	0.81	0.94	3,874	2.2E-16
	2-Archaea	0.37	0.88	233	3.0E-16
	3-Bacteria	0.26	0.85	182	9.6E-15
	4-Eukarya	0.12	0.76	108	9.3E-12

*Linear relationships were tested with the F statistics and coefficients of determination (R^2^)*.

Since Figures 4, 9 place proteomic growth within a temporal framework, combining those results with the growth and scaling patterns of Figure 2 confirm that the dynamic process of vocabulary growth of structural domains can be described in static terms with the Heaps law, with growth of database size measured as collection of FSF abundances of proteomes. This property matches the evolutionary growth of languages, derived from the analysis of hundreds of years of text corpora (two centuries in Petersen et al., 2012) showing the growth dynamic and the static scaling patterns of word innovation are linked. Our results also show that kernels of *total* and ABEV FSF vocabularies exist for the proteomes of each supergroup of life that are constant over billions of years. These kernels of FSFs frequently found in proteomes are complemented with a growing set of FSF vocabularies. However, as time progresses there is a slowdown of domain innovation that can be illustrated by the decreasing Heaps exponents of the power law regimes. This outcome probably stems from economies of scales manifesting at the molecular level, as we have shown for the combination of domains in multi-domain proteins following a MA law of decreasing returns (Shahzad et al., 2015). It also likely results from “semantic compression,” a process of compacting vocabulary with time by reducing language heterogeneity without affecting its semantics (conveying a same message with a smaller number of words, Chomsky, 1995; Sayood and Khalid, 2006).

While there are a number of Heaps-like scaling relationships in the vocabulary of genomes that appear universal, some reflecting the scaling of number of genes in different functional categories as a function of genome size (Molina and van Nimwegen, 2009), the link between dynamic and static properties of the models must always be confirmed with phylogenetic methods. We recently built global dynamic models for the evolution of structural domains that used birth-death differential equations with global abundances of domains as state variables without the need to capture the distribution of domains in proteomes (Tal et al., 2016). We fitted the models to data from ToDs assuming that only transitions present in the trees were possible between fold structures and that branches emerged directly from a trunk. We found that parameters of growth of domains within FSFs (FSF *reuse*) and diversification of FSFs (FSF *use*) showed emergent biphasic patterns with opposing trends, i.e., increases in FSF innovation were always counterbalanced by decreases in growth of FSF abundance, and vice versa, with the growth of the many more recent FSFs offsetting the growth of the older FSFs (Tal et al., 2016). Since the model is global and independent of the existence of proteomes, simulations suggest a frustrated and complex interplay of growth and diversification of domain structures in the protein world that emerges from organismal diversification but is not a consequence of proteome size. This complements the findings of proteome size mappings of evoPCO plots (Figure 9) and the links of a Heaps law with history that we have formalized.

### Phylogenetic tracings support the cellular origins of viral lineages

Our phylogenomic tracings support the primordial cellular origin of viruses and the gradual rise of molecular diversity in proteome evolution (Nasir and Caetano-Anollés, 2015). The first of the four regimes of the Heaps law (the kernel regime) corresponds to the viral group (Table 3) and phylogenetic tracings confirm that scaling is historical (Figures 4, 9). Comparative genomics provides additional evidence: the remarkably large number of *universal* FSFs that are widespread in cellular and viral proteomes (22% of *total* FSFs) and harbor ancient proteins associated with cell membranes supports the ancient domain kernel. Similarly, the existence of V_*abe*_ FSFs (*n* = 68) in archaeoviruses, bacterioviruses, and eukaryoviruses also indicates that viral lineages existed prior to cellular diversification. This pushes viral origins back to ancient cells harboring segmented RNA genomes (since viruses with these features were basal in our ToL, Nasir and Caetano-Anollés, 2015) from which modern viral lineages originated either via “escape” or “reduction” (Hendrix et al., 2000; Forterre, 2006; Holmes, 2011a; Forterre and Krupovic, 2012), albeit the reduction scenario was relatively better supported by our data and also by the discovery of giant viruses that overlap cellular endosymbionts and parasitic species in genome and particle sizes (La Scola et al., 2003; Philippe et al., 2013; Legendre et al., 2014, 2015) evolving in a similar way (Claverie and Abergel, 2013).

Historically, however, the origin of viral lineages prior to the ancestors of Archaea, Bacteria, and Eukarya has been taken with skepticism as viruses by definition must reproduce inside their cellular hosts and are tightly associated with proteins (i.e., capsids) thus requiring ribosome-encoding cells for reproduction. However, virus-early scenarios do not mean “virus-first” in evolution (as interpreted by Harish et al., 2016), but only prior to the last universal common ancestor of modern cells (Forterre, 2005). This ancestor itself had many cellular ancestors that should better be referred to as “ancient” or “primordial” cells. Indeed, fossil records have indicated existence of primordial cells early in evolution (Javaux et al., 2010; Wacey et al., 2011). In other words, a distinction between ancient and modern cells is necessary for broader understanding of virus-early scenarios and to overcome roadblocks preventing acceptance of viruses as major players in the evolutionary biology of cells. To quote Forterre (2016), “*The confusion between ‘cells’ and ‘modern cells’ (the descendants of the last universal common ancestor) is another major drawback in discussions about the origin of viruses”* (Forterre, 2016). Thus, our conjecture simply triggers atypical thinking about viral origins and evolution, which may be timely given how the discovery of giant viruses has broken multiple epistemological barriers (Claverie and Abergel, 2016).

Finally, viruses have been routinely considered as “pickpockets” of cellular genomes (Moreira and Lopez-Garcia, 2009). This claim however greatly underestimates virus-cell interactions and has been challenged by several independent analyses confirming the existence of an abundance of virus-specific genes in viral lineages (Daubin et al., 2003; Cortez et al., 2009) and from endogenous integrated viral-like elements in cellular genomes (Katzourakis and Gifford, 2010; Cornelis et al., 2012) suggesting that gene flow from viruses-to-cells likely exceeds gene transfer from cells-to-viruses (reviewed by Forterre, 2016, see also Claverie and Abergel, 2016). In brief, our evolutionary model is biphasic in nature and reconstructs an early “cell-like” phase in viral evolution distinguished from modern viral lineages. Interestingly, the cell-like phase in viral evolution can be restored today when viruses take over cellular machinery and produce viral factories that resemble cell-like organelles (Claverie, 2006) or when they endogenize cellular genomes either in the form of integrated elements or plasmids (Weiss, 2006; Holmes, 2011b).

### Synthesis

Here we show that Harish et al. (2016) failed to challenge the virus-early scenario that is supported by our phylogenomic data-driven retrodictive exploration (Nasir and Caetano-Anollés, 2015). Their claim that our rooting approach attracts the proteomes of organisms (and viruses) with small genomes to the base of rooted trees does not hold in light of our demonstrations because tree topology is established *prior* to rooting and character polarization. Furthermore, they asserted that our ToLs were rooted *a priori* with an indirect method and an outgroup taxon they interpreted as an ancestor, when in reality we root our ToLs *a posteriori* using a direct method that follows Weston's generality criterion (Weston, 1988, 1994). They utilized *total* FSF *use* as proxy for genome size while our phylogenomic data matrices optimize both *universal* FSF *use* and *reuse* during unrooted tree reconstruction. Their trees are not supported by tree metrics of any kind (in addition to several other inaccuracies) and are derived from a subset of our data matrices (representing only 16% of our taxa) that were selected (apparently) without a rationale to showcase desired topologies. In contrast, we show that proteome size tracings along historical evoPCO projections and ToLs derived from a universal biology of evolutionarily conserved protein folds not only controvert unfounded phylogenetic attractions but reveal a hidden interplay between protein fold innovation and abundance. This interplay holds true for simpler viruses and Archaea to more complex Bacteria and Eukarya. Remarkably, it materializes in a multi-regime Heap's law of vocabulary growth (Figure 2) that makes explicit the axiom of historical continuity that is a cornerstone of evolutionary thinking and ToL reconstruction.

## Materials and methods

Phylogenomic data and reconstruction methods follow Nasir and Caetano-Anollés (2015). In brief, a census of structural domains in proteomes defined a phylogenetic data matrix of FSF *reuse*, which was normalized, encoded and used to build most parsimonious phylogenetic trees using PAUP^*^ (Swofford, 2002). Optimal trees were rooted using Weston's generality criterion implemented with the Lundberg method (Lundberg, 1972), which polarizes character state change without specification of an outgroup or ancestor. Rogue taxa identification and TII calculations were performed using RogueNaRok (Aberer et al., 2013). LS measurements and Explicitly Agree (EA) similarities were calculated with RadCon (Thorley and Page, 2000). EvoPCO analysis was performed using Excel XLSTAT plugin as described in Nasir and Caetano-Anollés (2015). Since proteomic make up involves a collective of FSFs of different ages, we use *nd* values of age derived from a ToD to transform an FSF occurrence (FSF *use*) matrix into an FSF occurrence^*^(1−*nd*) matrix. This makes it possible to study a multidimensional space of “reverse” evolutionary ages of domains without losing information of FSF of very ancient origin or introducing biases from FSF absences. Euclidean distances describing dissimilarities between proteomes were calculated and the distance matrices were used to calculate the first three principal coordinates describing maximum variability in data. These three most significant loadings described how FSF parts contributed to the history of proteome systems.

## Author contributions

AN, KK and GCA contributed to the design, experimentation, and analysis of the study, drafted, edited, improved, and finalized the manuscript.

### Conflict of interest statement

The authors declare that the research was conducted in the absence of any commercial or financial relationships that could be construed as a potential conflict of interest. The reviewer CH and handling Editor declared their shared affiliation, and the handling Editor states that the process nevertheless met the standards of a fair and objective review.
