# Supplementary material for: Phylogenetic Tracings of Proteome Size Support the Gradual Accretion of Protein Structural Domains and the Early Origin of Viruses from Primordial Cells
Source: Front Microbiol. 2017 Jun 23;8:1178. doi: 10.3389/fmicb.2017.01178 (PMC5481351; doi:10.3389/fmicb.2017.01178)
Supplement: Supplementary file 1 [file DataSheet1.PDF]

Supplementary Material

Phylogenetic Tracings of Proteome Size Support the Gradual Accretion of Protein Structural Domains and the Early Origin of Viruses from Primordial Cells

Arshan Nasir[1], Kyung Mo Kim[2], Gustavo Caetano-Anollés[3*]

*** Correspondence:** Gustavo Caetano-Anollés: gca@illinois.edu

1 Supplementary Figures and Tables

1.1 Supplementary Figures

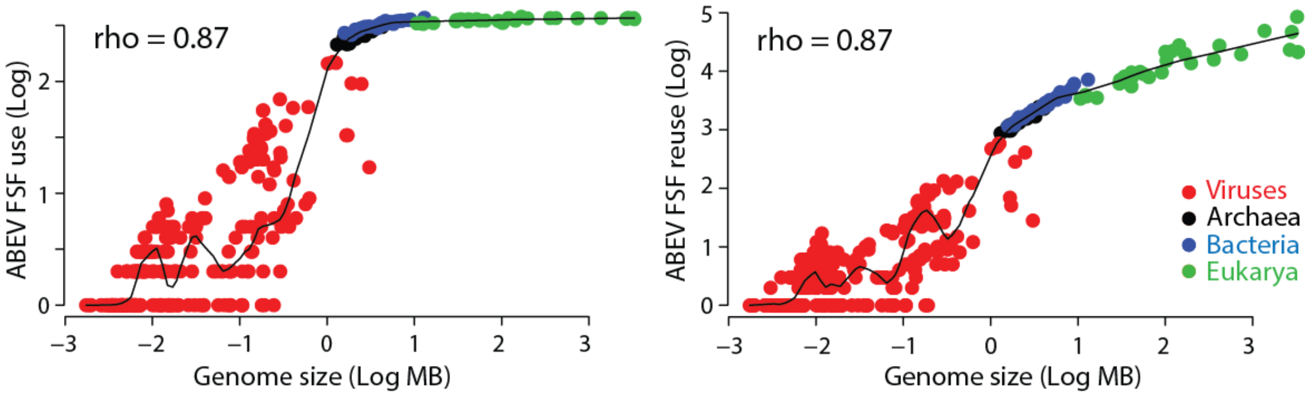

Figure S1. FSF *use* (occurrence) and *reuse* (abundance) reveal complex relationships with genome size. Scatter plots reveal correlations between genome size (mega bases, MB) and *universal* ABEV FSF *use* (left panel) and ABEV FSF *reuse* (right panel) for 255 viruses (red), 30 Archaea (black), 31 Bacteria (blue), and 28 Eukarya (green). The black lines describe the nature of the relationship, as determined by the Locally Weighted Regression Scatter Plot Smoothing (LOWESS) method, which obtains a smoothed curve by fitting successive regression functions ($q = 0.1$, $i = 100$).

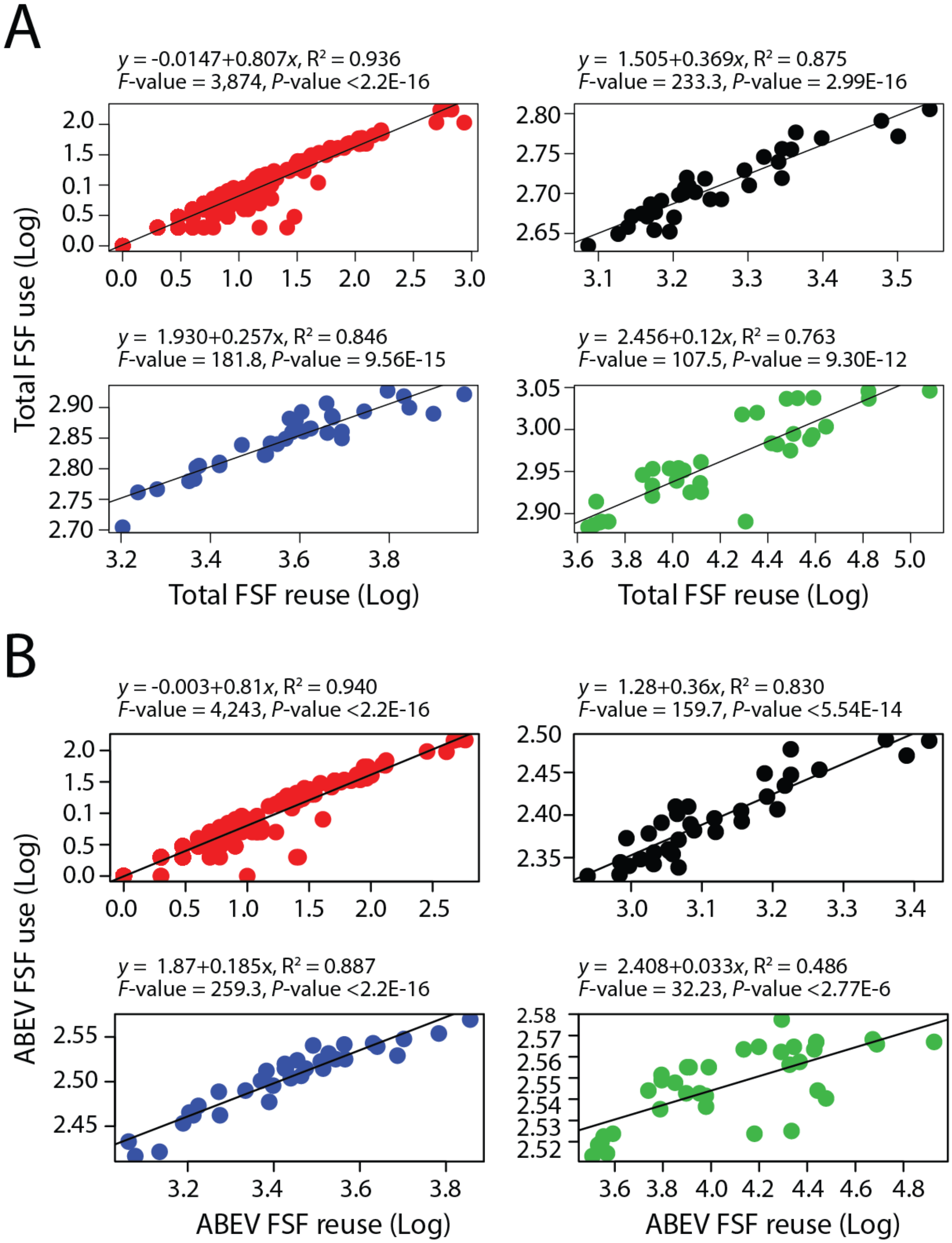

Fig S2. FSF *use* and *reuse* log-log plots reveal four distinct power law regimes for the *total* (A) and *universal* (B) FSF sets of the proteomes of viruses (red), Archaea (black), Bacteria (blue) and Eukarya (green). The black lines describe the fitted regression function with equations listed at the top, including F statistics and coefficients of determination (R^2).

1.2 Supplementary Tables

Table S1. V_{abe} FSFs are not restricted to eukaryoviruses. The list of 68 V_{abe} FSFs along with SCOP IDs, SCOP *concise classification strings (css)* and the number of archaeoviruses (AVs), bacterioviruses (BVs), and EVs that encode the FSF. The last column indicates whether the FSF was detected in *Acanthamoeba polyphaga mimivirus* proteome or not.

Table S2. List of organisms and viruses used in our study. FSF *use* and *reuse* values are listed for both *total* and *universal* sets.

Table S3. Leaf stability and similarity measurements. LS Maximum, LS Difference, LS Entropy, and EA Similarity statistics are given for the control set (i.e. 5 taxa each from the four supergroups) and for each set of permutations described in Fig 7. Values in red indicate decreased leaf stability compared to the control and were more common in *R. prowazekii* permutations. Asterisks are distributions significantly different from control (C) (Wilcoxon rank sum test, two-tailed, $P < 0.01$).
