# Supplementary material for: Phylogenetic Tracings of Proteome Size Support the Gradual Accretion of Protein Structural Domains and the Early Origin of Viruses from Primordial Cells
Source: Front Microbiol. 2017 Jun 23;8:1178. doi: 10.3389/fmicb.2017.01178 (PMC5481351; doi:10.3389/fmicb.2017.01178)
Supplement: Supplementary file 2 [file Table1.PDF]

Table S1. V_{abe} FSFs are not restricted to eukaryoviruses (EVs). The list of 68 V_{abe} FSFs along with SCOP IDs, SCOP *concise classification strings (css)* and the number of archaeoviruses (AVs), bacterioviruses (BVs), and EVs that encode the FSF. The last column indicates whether the FSF was detected in the mimivirus or not.

SCOP ID	SCOP (*css*)	FSF Description	Count (AV)	Count (BV)	Count (EV)	Mimivirus? (Yes, No)
53098	c.55.3	Ribonuclease H-like	11	701	302	Yes
56672	e.8.1	DNA/RNA polymerases	13	580	1372	Yes
50249	b.40.4	Nucleic acid-binding proteins	4	478	83	Yes
48168	a.98.1	R1 subunit of ribonucleotide reductase, N-terminal domain	5	181	72	Yes
56091	d.142.2	DNA ligase/mRNA capping enzyme, catalytic domain	1	281	95	Yes
52540	c.37.1	P-loop containing nucleoside triphosphate hydrolases	51	1032	1184	Yes
51998	c.7.1	PFL-like glycyl radical enzymes	8	284	131	Yes
57783	g.41.3	Zinc beta-ribbon	4	250	70	Yes
52980	c.52.1	Restriction endonuclease-like	16	312	140	Yes
56399	d.166.1	ADP-ribosylation	2	73	19	Yes
49842	b.22.1	TNF-like	1	19	7	Yes
52799	c.45.1	(Phosphotyrosine protein) phosphatases II	8	12	87	Yes
47413	a.35.1	lambda repressor-like DNA-binding domains	2	546	5	Yes
88723	c.120.1	PIN domain-like	2	162	56	Yes
56235	d.153.1	N-terminal nucleophile aminohydrolases (Ntn hydrolases)	1	44	17	Yes
46565	a.2.3	Chaperone J-domain	2	20	80	Yes
46955	a.6.1	Putative DNA-binding domain	1	144	5	Yes
46785	a.4.5	Winged helix DNA-binding domain	27	173	31	Yes
52141	c.18.1	Uracil-DNA glycosylase-like	2	22	101	Yes
53448	c.68.1	Nucleotide-diphospho-sugar transferases	5	103	68	Yes
54060	d.4.1	His-Me finger endonucleases	6	443	18	Yes
50998	b.70.1	Quinoprotein alcohol dehydrogenase-like	1	2	7	Yes
53300	c.62.1	vWA-like	8	99	10	Yes
53335	c.66.1	S-adenosyl-L-methionine-dependent methyltransferases	13	383	198	Yes
52833	c.47.1	Thioredoxin-like	2	289	53	Yes
55979	d.131.1	DNA clamp	3	114	84	Yes
56300	d.159.1	Metallo-dependent phosphatases	12	405	23	Yes
47954	a.74.1	Cyclin-like	1	1	28	Yes
55608	d.95.2	Homing endonucleases	9	67	12	Yes
52402	c.26.2	Adenine nucleotide alpha hydrolases-like	3	67	18	Yes
52374	c.26.1	Nucleotidylyl transferase	1	71	10	Yes

55729	d.108.1	Acyl-CoA N-acyltransferases (Nat)	3	86	29	Yes
109604	a.211.1	HD-domain/PDEase-like	1	78	10	Yes
52317	c.23.16	Class I glutamine amidotransferase-like	1	3	20	Yes
117892	d.43.2	Band 7/SPFH domain	6	58	7	Yes
56784	c.108.1	HAD-like	1	202	101	Yes
56349	d.163.1	DNA breaking-rejoining enzymes	28	365	116	Yes
55874	d.122.1	ATPase domain of HSP90 chaperone/DNA topoisomerase II/histidine kinase	4	105	33	Yes
48019	a.80.1	post-AAA+ oligomerization domain-like	3	3	15	Yes
64496	d.285.1	DNA-binding domain of intron-encoded endonucleases	2	79	9	Yes
47794	a.60.4	Rad51 N-terminal domain-like	6	3	6	Yes
81301	d.218.1	Nucleotidyltransferase	1	49	16	Yes
48695	a.138.1	Multiheme cytochromes	1	6	3	Yes
51735	c.2.1	NAD(P)-binding Rossmann-fold domains	2	33	44	Yes
102114	c.1.28	Radical SAM enzymes	4	66	4	Yes
101386	a.204.1	all-alpha NTP pyrophosphatases	3	271	10	Yes
48452	a.118.8	TPR-like	3	6	11	Yes
101898	b.68.9	NHL repeat	1	2	4	Yes
53756	c.87.1	UDP-Glycosyltransferase/glycogen phosphorylase	18	19	87	Yes
51283	b.85.4	dUTPase-like	12	133	175	No
57667	g.37.1	beta-beta-alpha zinc fingers	15	2	13	No
69796	d.207.1	Thymidylate synthase-complementing protein Thy1	13	202	12	No
49899	b.29.1	Concanavalin A-like lectins/glucanases	19	84	17	No
48371	a.118.1	ARM repeat	3	117	14	No
51126	b.80.1	Pectin lyase-like	2	168	44	No
58100	h.4.4	Bacterial hemolysins	1	18	9	No
50494	b.47.1	Trypsin-like serine proteases	1	10	475	No
49265	b.1.2	Fibronectin type III	2	145	21	No
102405	c.129.1	MCP/YpsA-like	1	40	4	No
56747	d.264.1	Prim-pol domain	3	98	63	No
56563	d.183.1	Major capsid protein gp5	8	410	2	No
50939	b.68.1	Sialidases	1	9	40	No
88659	a.4.13	Sigma3 and sigma4 domains of RNA polymerase sigma factors	7	116	6	No
55620	d.96.1	Tetrahydrobiopterin biosynthesis enzymes-like	3	13	2	No
47781	a.60.2	RuvA domain 2-like	6	22	2	No
48150	a.96.1	DNA-glycosylase	1	6	3	No
50199	b.40.1	Staphylococcal nuclease	2	40	4	No
50965	b.69.1	Galactose oxidase, central domain	1	2	6	No
